# Interval and Ratio Scaling of Spectral Audio Descriptors

**DOI:** 10.3389/fpsyg.2022.835401

**Published:** 2022-03-30

**Authors:** Savvas Kazazis, Philippe Depalle, Stephen McAdams

**Affiliations:** Schulich School of Music, McGill University, Montreal, QC, Canada

**Keywords:** timbre, audio descriptors, psychological scales, perception, psychoacoustics, audio features, partition scaling, global psychophysics

## Abstract

Two experiments were conducted for the derivation of psychophysical scales of the following audio descriptors: spectral centroid, spectral spread, spectral skewness, odd-to-even harmonic ratio, spectral deviation, and spectral slope. The stimulus sets of each audio descriptor were synthesized and (wherever possible) independently controlled through appropriate synthesis techniques. Partition scaling methods were used in both experiments, and the scales were constructed by fitting well-behaving functions to the listeners' ratings. In the first experiment, the listeners' task was the estimation of the relative differences between successive levels of a particular audio descriptor. The median values of listeners' ratings increased with increasing feature values, which confirmed listeners' abilities to estimate intervals. However, there was a large variability in the reliability of the derived interval scales depending on the stimulus spacing in each trial. In the second experiment, listeners had control over the stimulus values and were asked to divide the presented range of values into perceptually equal intervals, which provides a ratio scale. For every descriptor, the reliability of the derived ratio scales was excellent. The unit of a particular ratio scale was assigned empirically so as to facilitate qualitative comparisons between the scales of all audio descriptors. The construction of psychophysical scales based on univariate stimuli allowed for the establishment of cause-and-effect relations between audio descriptors and perceptual dimensions, contrary to past research that has relied on multivariate stimuli and has only examined the correlations between the two. Most importantly, this study provides an understanding of the ways in which the sensation magnitudes of several audio descriptors are apprehended.

## 1. Introduction

Audio features have been widely used in timbre research for explaining quantitatively the dimensions of timbre spaces (Grey and Gordon, 1978; Iverson and Krumhansl, 1993; McAdams et al., 1995; Lakatos, 2000), affective ratings (Laurier et al., 2009; Farbood and Price, 2017; McAdams et al., 2017), and the perceptual similarity of short music clips (Siedenburg and Müllensiefen, 2017). Most often, the spectral features are derived from statistical computations on a spectrogram, whereas the temporal features are usually extracted from the raw waveform. The time-series of these features, derived from a frame-by-frame analysis on the spectrogram, are then compressed through summary statistics into single numbers, which are presumed to serve as the spectrotemporal imprint of a stimulus and thereby designate aspects of its timbre. The systematic development of such features started with the work of Krimphoff et al. (1994) for explaining quantitatively the perceptual dimensions of Krumhansl's (1989) timbre space. The set of features started expanding with the appearance of the MPEG-7 standard (ISO/IEC, 2002), according to which the audio features would be termed *audio descriptors*. These two terms will be used interchangeably in the present document.

Most of the past research on timbre psychophysics has focused on determining acoustic correlates of perceptual dimensions derived from multidimensional scaling of dissimilarity ratings, in order to quantify the ways in which we perceive sounds to differ. However, there is little empirical evidence to date demonstrating that acoustic features derived from correlational analysis *causally* correspond to psychological dimensions. Most importantly, even for cases in which the causality has been verified, there is almost no research on the sensation magnitudes of such acoustic features. The aim of the present study is to provide an understanding of how the sensation magnitudes of timbre-related audio features are apprehended through the construction of psychophysical scales. In addition, audio descriptors have been widely used as predictor variables in statistical regression models for interpreting and predicting listeners' responses on a variety of tasks that relate to timbre. However, the physical values of these predictors may lead to misinterpretations about their perceptual significance on a particular task. The derived scales from the present study may allow timbre researchers to use perceptually informed values of spectral descriptors as predictors in their statistical models that may lead to more sustainable conclusions and accurate interpretations in terms of perception. In the following sections, we present the experiments used to derive the perceptual interval and ratio scales of the following audio features (a mathematical formulation of these descriptors can be found in Peeters et al., 2011).

*Spectral centroid* (also known as the *spectral center of gravity*): the frequency weighted average of the (power) spectrum; related to auditory brightness. Low centroid values indicate a dark sound and high values a bright sound. However, it also increases in the presence of noise, and it tends to fluctuate to a great extent during the transient regions of sound events. In many cases it is also correlated with fundamental frequency and pitch.

*Spectral spread* (also known as *instantaneous bandwidth*, and *spectral standard deviation*): the standard deviation of the (power) spectrum around the spectral centroid. High values indicate a rich spectrum.

*Spectral skewness*: It is defined as the third statistical moment of the spectrum; a measure of the asymmetry of the spectrum around the spectral centroid. Zero skewness indicates a symmetric distribution around the spectral centroid. Negative skewness indicates more energy at lower frequencies whereas positive skewness indicates more energy at higher frequencies. Most instrument sounds (rich in overtones or harmonics) exhibit positive skewness. However, mixtures of different sound sources may exhibit nearly symmetric distributions.

*Odd-to-Even ratio*: the ratio of energies of the odd to the even harmonics. High odd-to-even ratio indicates more energy at the odd harmonics (e.g., the clarinet) and often results in “hollow” sounds. A lower odd-to-even ratio indicates a smoother spectrum and a “fuller” sound (e.g., the trumpet). The square and triangle waveforms used in analog synthesis also have a high odd-to-even ratio (energy only in the odd harmonics), whereas the richer spectrum of the sawtooth waveform has a lower ratio (energy in both the odd and even harmonics).

*Harmonic Spectral Deviation*: similar to the odd-to-even ratio but can also be applied to inharmonic sounds. It is defined as the normalized sum of differences between the magnitude of a center frequency and the average magnitude of itself and its two neighboring frequencies. It is a measure of “jaggedness” of the spectrum. Some sounds tend to sound “nasal” as spectral deviation increases (depending on the fundamental frequency). However, extremely low spectral deviation indicates a flat spectrum which often sounds “harsh” (depending on the spacing of the overtones).

*Spectral Slope*: measures the slope of the spectrum. This descriptor is an approximation of the spectral shape computed by a linear regression over the spectral magnitudes. Most instrument sounds exhibit negative spectral slopes because the energy of the upper harmonics decreases by harmonic number. The fundamental waveforms used in analog synthesis also have different slopes. For instance, the (negative) slope of a triangular waveform is twice as steep compared to that of a square waveform and is often described as “warmer” than the square waveform. A spectral slope close to zero indicates a flat spectrum (equal energy at all frequencies) and is related to “noisy” regions. In many cases, spectral slope is correlated with spectral centroid.

As previously mentioned, timbre research has mainly relied on correlational analysis between audio features and listeners' perceptions, but there have been a few attempts to establish causal relations between psychological and acoustic dimensions. One of these attempts is the confirmatory study of Caclin et al. (2005), who validated with synthesized stimuli the salience of attack time, spectral centroid, and the odd-to-even harmonic ratio, but not spectral flux for explaining dissimilarity ratings. Another study is from Almeida et al. (2017) who attempted with synthesized stimuli to derive a ratio scale of brightness as a function of spectral centroid, albeit within a limited range of 1.46 octaves and at a single fundamental frequency of 500 Hz. In fact, neither of the aforementioned studies evaluated directly the spectral centroid, but rather the spectral slope, which co-varies with spectral spread, skewness, and under certain circumstances is linearly dependent on spectral centroid. More recently, Kazazis et al. (2021) validated through ordinal scaling with synthesized stimuli several audio features by controlling each spectral feature independently of the rest, thus isolating the effect that each feature had on the stimulus rankings. The results of those experiments have served as the basis for the present study, because there was no prior evidence that stimuli varying along a particular audio feature could be perceived on an ordinal scale, the existence of which is a prerequisite for constructing perceptual interval and ratio scales.

Different experimental procedures are needed for testing different scales: an ordinal scale indicates whether listeners are able to rank order the stimuli; an interval scale, whether they can judge the relative size of intervals between stimuli; and, a ratio scale, whether ratios between stimuli can be perceived. However, the most informative scale is the ratio scale, which satisfies all the criteria of an interval scale, but also enables the derivation of ratios between stimuli. In other words, the ratio scale subsumes the interval scale and the experimental procedure should devise operations for determining the following relations among stimuli (Stevens, 1946): equality; rank order; equality of intervals; and, equality of ratios.

There are some important methodological considerations that need to be taken into account before designing experiments for deriving either interval or ratio scales of audio features. Interval and ratio scaling methods are part of “global psychophysics” rather than “local psychophysics”, where the aim is usually to derive just noticeable differences (JNDs) among stimuli, which do not predict the results of global psychophysical experiments. A psychophysical experiment is said to be global if the extreme stimuli of a stimulus set are almost perfectly identified in a two-stimulus absolute identification design (Luce and Krumhansl, 1988). This has certain implications in the construction and selection of appropriate stimuli, which will be discussed further in the next section. The methods used for constructing psychophysical interval and ratio scales, based on direct estimations of subjective magnitudes, can be classified into two general categories. In *magnitude estimation* (or *production*), listeners are instructed to assign numbers of their choice to stimuli so as to reflect subjective ratios in relation to a reference stimulus (or *standard*), which is usually located in the middle of the presented range of values. In *category scaling* (or *difference estimation*), the lower and upper limits of the response scales are defined, and listeners are instructed to assign scale values along the continuum between the extremes so as to preserve subjective differences (or psychological distances) between stimuli. Irrespective of the method used, in most scaling experiments, the physical attributes of study can be easily explained and identified by the listeners and are often associated with a perceptual correlate such as loudness or pitch. One of the issues and challenges that arise in psychophysical scaling of audio features is that the experimenter cannot describe with clarity and in simple terms the attribute under study to the listeners, without resorting to a purely technical formulation of a particular audio feature, which in most cases will not be understandable by “naïve” participants (e.g., musicians without a physics background). Stevens (1946) suggests that this difficulty often arises because audio features are measured on *derived* physical scales constructed by mathematical functions of certain magnitudes, which themselves are derived from *fundamental scales* for which a perceptual correlate can be more easily found (e.g., loudness for intensity). In the present case, which deals with audio features, the fundamental scales are represented by fundamental frequency and the magnitude-frequency pairs of spectral components.

## 2. Justification of the Experimental Procedure

In a first attempt to derive ratio scales, we designed a pilot experiment based on magnitude estimation, in which the largest effects are produced by the range of stimuli, the distance from threshold (if a threshold exists for a particular feature), and the degree of freedom given to listeners for choosing the lowest and the highest number for their responses (Poulton, 1968). Several sets of stimuli for the different audio descriptors were constructed by controlling the magnitude of a particular descriptor while the rest were kept constant (see the Methods subsection). The standard was positioned in the middle of a particular stimulus set (in terms of descriptor magnitudes) and listeners were limited to *one judgement per stimulus*, which reduces the biases due to range and spacing of stimuli (Stevens, 1971). In each trial, listeners were presented with a single stimulus followed by the standard (reference stimulus) and were instructed to assign a number of their choice that reflected the subjective magnitude ratio between the two. Throughout the trials, the standard was always positioned in the middle of a particular stimulus set, and only a single stimulus was presented in each trial (instead of asking listeners to make simultaneous judgments on two or more stimuli with respect to the standard), in order to reduce the biases due to range and spacing of stimuli (Stevens, 1971). In addition, there were no limitations on the available set of numbers used as responses for both the stimulus and the standard other than the requirement of being positive, because any such limitations have been shown to increase the bias in magnitude estimation experiments (Hellman and Zwislocki, 1961). We consider this experiment to be unsuccessful, because the sensation magnitudes were poorly apprehended. In other words, listeners' judgments were inconsistent (within and across participants) when estimating the magnitudes of stimuli independently and with respect to the standard. The averaged magnitude estimations (across and within participants) indicated that the stimulus sets could not even be rank-ordered since there was no consensus on the magnitudes of the constituent stimuli. These results seemed paradoxical because it was known in advance that the stimulus sets could be perfectly rank ordered (Kazazis et al., 2021) when the judgments were made simultaneously (i.e., across all stimuli of a particular stimulus set). These results can be mainly attributed to the experimental design. First of all, for some audio features, the stimuli were hardly discriminable leading to poor independent judgments when each stimulus was presented in isolation from the rest. In some other cases, stimuli were very distant from the standard (in terms of physical magnitude), which is considered to be a source of bias in magnitude estimation experiments, because although stimuli near to the standard are judged relative to the standard, stimuli far from the standard are not (Gescheider and Hughson, 1991). Most importantly, listeners were not given any indication of which attribute they were judging between presented stimuli other than the written instruction “… according to any criteria that differentiate them the most.” Due to the nature of the stimuli and because of the reasons related to the psychophysical scaling of audio features described above, the attribute of study could only have been identified by the listeners if the experimental design had allowed for the discovery of invariances among stimuli through the exploration of a particular stimulus set, instead of a presentation of stimuli in isolation. Finally, experiments based on magnitude estimation place a heavy load on listener's memory and given the unfamiliarity of the listeners with the presented stimuli, this was considered to be an additional reason for which the magnitudes in this experiment were poorly apprehended. This last implication could have been avoided if the method of *absolute magnitude estimation* (Hellman and Zwislocki, 1961) had been used instead, in which listeners match numbers to stimuli without the presentation of a standard, and independently of the previous matches. However, an experimental design based on this method would not have been able to overcome the above-mentioned hurdles and provide positive results, mainly because it is difficult to make absolute judgments on timbral attributes.

In a second pilot experiment, the task was the same as in the experiment described at the beginning of the previous paragraph except that in this one, listeners were asked to make *simultaneous* judgments in each trial with respect to a standard on the whole stimulus set of a particular audio descriptor. More specifically, in each trial listeners were presented with the stimulus set in the form of sound boxes (on screen). Clicking on each soundbox would trigger a particular stimulus followed by the standard. Therefore, listeners could explore the range of each stimulus set by triggering all the sound boxes prior to making their judgments. There was no limitation on the number of times a soundbox could be triggered, and the stimuli were randomized (i.e., stimuli were not presented in an ascending or descending series of physical magnitude). However, the participants were instructed to make magnitude estimations between a particular stimulus and the standard, rather than making simultaneous comparisons across the stimuli. As in the previous experiment, they could assign numbers of their choice to both the standard and the stimuli. From a methodological point of view, this design is a compromise between category scaling (or *difference estimation*) and magnitude estimation, but it has also been shown to lead to a compromise between the derived scales of the two (Montgomery, 1975). Although this design allowed listeners to explore a range of stimuli, and thus identify the attribute of study more effectively than the previous experiment, this design had its own pitfalls. The most important bias resulted from the spacing of stimuli, which magnitude estimation methods aim to control for by restricting listeners to perform one judgment per stimulus. In other words, the derived scales may not be generalizable, in a sense that a different spacing of stimulus values might have led to different scales. However, there were also some practical issues, which limited the credibility of the results. Given that the order of presentation was random, and that a large number of stimuli were presented in a single trial, some listeners might only have focused on rank ordering, when verifying their judgments by listening to the stimuli sequentially and indexing them according to their magnitude estimations, instead of making more accurate judgments between the stimuli and the standard. In that case, it might be expected that the outcome of this experiment would have been the same as the results provided by Kazazis et al. (2021) whereas the main aim of this experiment was to provide additional insights into how the sensation magnitudes of descriptor values are apprehended. In addition, some listeners complained about the difficulty of the task, and reported that they were performing comparisons across all the presented stimuli, although it was clearly stated in the instructions that the comparisons should only be performed between a single stimulus and the standard. Such operations considerably increased the cognitive load of the listeners, which might have had a strong impact on the accuracy of their judgments.

Because of all the above-mentioned issues of each experimental design, we employed partition scaling methods (Stevens, 1975) for constructing both interval and ratio scales of audio features. These methods have been successfully used in the past, such as for the derivation of the Mel scale for pitch (Stevens and Volkmann, 1940). This manuscript is organized as follows. In section 3, we present the synthesis processes used to construct the stimulus set for each audio feature and the experiment for the derivation of interval scale measurements. Given the lack of previous knowledge of JNDs on these audio features, this experiment could be considered as a confirmatory experiment on whether listeners are actually able to perceive intervals before proceeding to the next experiment with its additional operations needed for deriving ratio scales (described in section 4). Finally, in section 5, we present concluding remarks on the validity of the obtained results and implications for timbre perception.

## 3. Experiment 1: Interval Estimation

The aim of this experiment was to investigate whether listeners perceive intervals of audio features and the construction of interval scales. The listeners' task was the estimation of the relative differences between successive levels of a particular audio feature, and thus this experiment provided interval scale measurements.

### 3.1. Method

#### 3.1.1. Participants

Twenty-five participants, 11 female, 13 male, and 1 “prefer not to answer”, with a median age of 23 years (range: 18–40) were recruited from the Schulich School of Music, McGill University. All of them were self-reported amateur or professional musicians with formal training in various disciplines such as performance, composition, music theory, and sound engineering. Participants were compensated for their time. The study was certified for ethical compliance by the McGill University Research Ethics Board II. Before the experiment, participants had to sign an informed consent form. Afterwards, they passed a pure-tone audiometric test at octave-spaced frequencies from 125 Hz to 8 kHz (Martin and Champlin, 2000; ISO 389-8, 2004) and were required to have thresholds at or below 20 dB HL to proceed to the experiment.

#### 3.1.2. Stimuli and Presentation

Several sets consisting of synthetic sounds were created by independently controlling the values of several spectral audio features in the synthesis process. For spectral centroid, spread, and skewness, all the spectral manipulations were applied to a flat harmonic spectrum (harmonics set at equal amplitude) with a fundamental frequency (f0) of 120 Hz and harmonics up to Nyquist limit. For spectral slope and deviation, as well as odd-to-even harmonic ratio, separate sound sets were synthesized at f0s of 120, 300, and 720 Hz with the number of harmonics ranging from 9 to 16, depending on the feature. Following the spectral manipulations, the stimuli were synthesized in Matlab version R2015b (The MathWorks, Inc., Natick, MA) using additive synthesis at a sampling frequency of 44.1 kHz with 16-bit amplitude resolution. The peak amplitude of the waveforms was normalized to 0.5 and the duration was set to 600 ms, gated with 10-ms raised-cosine ramps. All stimuli were loudness normalized according to the algorithm of Moore et al. (1997) and further adjusted by the authors because it was observed that the algorithm overestimated the loudness of sounds that had most of their energy centered at high frequencies.

For each feature, and for a particular spectral centroid or f0, the stimuli were presented in three different sequences of feature values between fixed anchor values, under the constraint that the values of two successive stimuli should be different for each sequence. The spacing of stimulus values presented in each sequence was based on the results of Kazazis et al.'s (2021) ordinal scaling experiment, in which listeners' confusions between successive stimuli were identified. This allowed for a supraliminal stimulus set within each sequence. In the following subsections we present the synthesis methods used for the construction of stimuli that led to independent control of the values of each feature. Table 1 lists the ranges of all feature values computed on a number of sounds generated to test a particular feature. As is evidenced by this table, most feature values within a particular stimulus set remain relatively constant or vary within a very narrow range compared to the ranges of feature values according to which the stimulus set was generated. The most notable exceptions are the stimulus sets of spectral slope and odd-to-even ratio. This is because, spectral slope naturally covaries with spectral centroid, spread, and skewness, and resulted in a greater skewness range than the stimulus set of spectral skewness due to the constraints imposed in the sound synthesis process that are outlined in the following subsections. In a similar way, the odd-to-even ratio is directly related to the computation of spectral deviation, and its respective stimulus set had a greater range of spectral deviation than the dedicated stimulus set of spectral deviation. The stimuli are provided as Supplementary Material.

**Table 1:** Ranges of feature values within designated stimulus sets.

	**Feature ranges**					

**Stimulus Sets (# sounds)**	**Centroid (Hz)**	**Spread (Hz)**	**Skewness**	**Odd-to-Even Ratio**	**Deviation**	**Slope (dB/octave)**
Centroid (505)	**[1642, 9560]**	[479, 480]	[0.00, 0.02]	[1.00, 1.00]	[0.00, 0.00]	-
Spread (100)	[5600, 5600]	**[181, 1439]**	[0.00, 0.00]	[1.00, 1.00]	[0.00, 0.00]	-
Skewness (97)	[5600, 5600]	[1079, 1080]	**[0.88, 0.96]**	[1.00, 1.00]	[0.00, 0.00]	-
Odd-to-Even Ratio (349)	[1260, 1500]	[768, 848]	[0.00, 0.21]	**[0.25, 1250.00]**	[0.00, 0.11]	[–11.67, –2.62]
Deviation (265)	[1723, 2550]	[1292, 1396]	[0.00, 0.28]	[1.00, 1.19]	**[0.00, 0.06]**	[0.00, –5.04]
Slope (349)	[332, 2082]	[134, 785]	[–1.04, 6.68]	[1.25, 15.05]	[0.00, 0.03]	**[–24.00, 5.44]**

**Figure 1:**
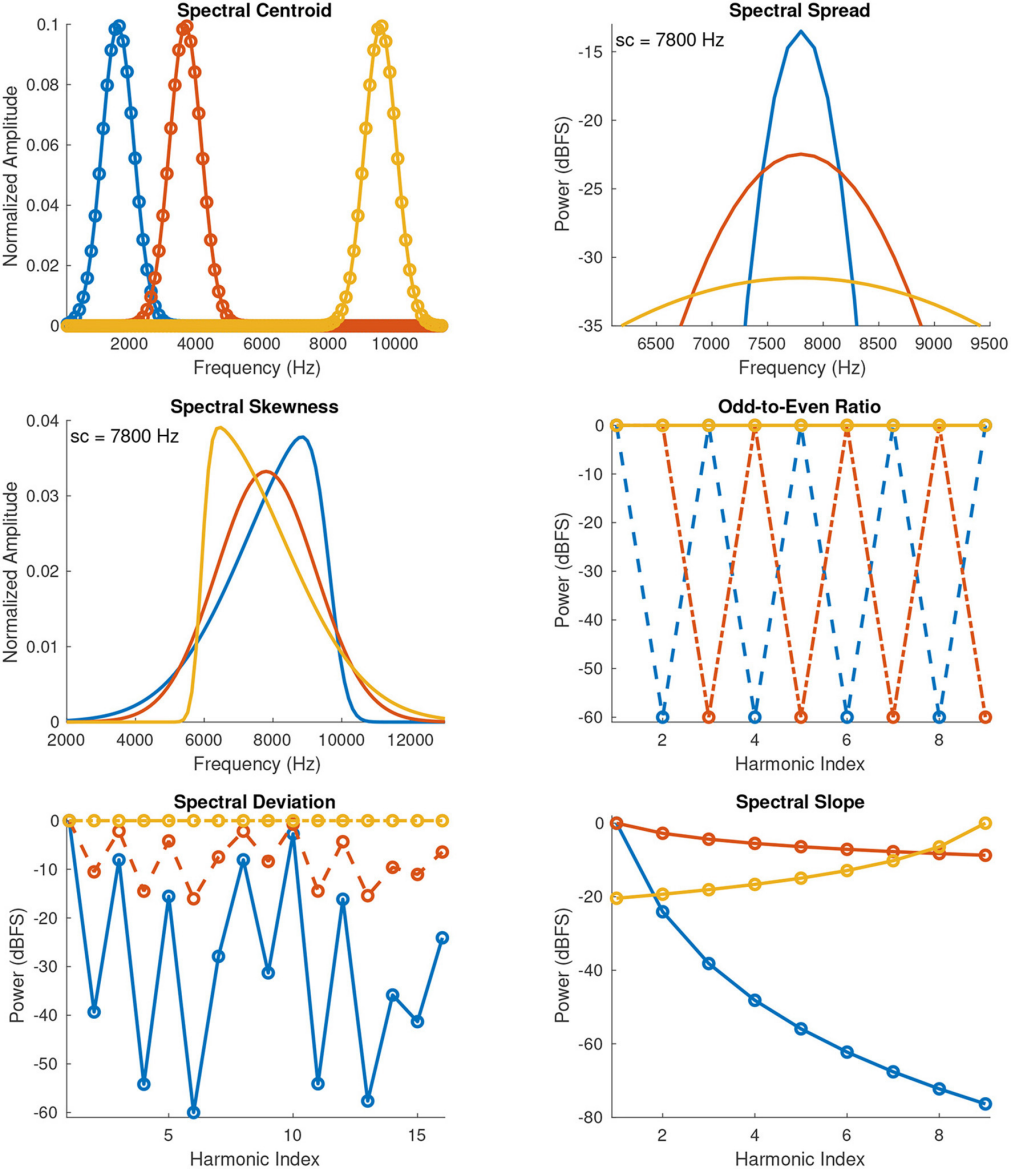
Spectral envelopes of anchor stimuli used in Experiments 1 and 2 (blue and yellow curves), and of mid-point stimuli (red curve) used in the rating scale of Experiment 2. The spectral envelopes of the mid-point stimuli correspond to the middle sound of each stimulus set reported in Table 1. Dots indicate harmonics (for the spread and skewness plots the dots are omitted for display purposes). sc, spectral centroid.

*The ranges of feature values according to which each stimulus set was generated are shown in bold. The number of sounds on which the feature values were computed are shown inside parenthesis. The reported ranges for the spectral spread and skewness stimulus sets were computed on stimuli with 5600-Hz spectral centroid. The value of spectral slope on bell-shaped spectral amplitudes is meaningless (cf. Figure 1)*.

##### 3.1.2.1. Spectral Centroid

The stimuli of these sound sets were constructed by shaping the flat harmonic spectrum described above to follow a normal probability mass function that enabled the construction of spectra with different centroids (means), for a given spread (standard deviation) and zero skewness (Figure 1). Note that the actual computed values (in discrete frequency) differ slightly from the theoretical values (calculated on continuous frequency) used in the synthesis processes due to round-off errors. The amount of spectral spread was set to 480 Hz (four times the f0), which for the f0 at 120 Hz allowed for a minimum centroid of 1,640 Hz and a fixed bandwidth of 9 harmonics for each stimulus's spectrum. It should also be noted that the harmonic spacing of the components ensured a (virtual) pitch percept at the f0. The spectral centroid values (in kHz) presented in each sequence (*Seq*.) were the following:

*Seq.1*: {1.64, 2.28, 3.68, 6.20, 9.56}

*Seq.2*: {1.64, 2.88, 3.68, 4.76, 9.56}

*Seq.3*: {1.64, 1.80, 3.68, 8.20, 9.56}

##### 3.1.2.2. Spectral Spread

The normal distribution was again used for constructing stimuli with fixed centroids, zero skewness and variable spectral spreads, by precisely controlling its bandwidth (Figure 1). The range of the allowable spreads in the synthesis process was constrained by the centroid and f0 used in each stimulus set, as well as the spacing resolution of the harmonics. Three sound sets were constructed with centroids centered at 1,640, 5,600, and 7,800 Hz that allowed for maximum spreads (with respect to the f0) of 480, 1,440, and 1,800 Hz, respectively. For each of these sound sets (i.e., for each of the three spectral centroids, respectively), the spectral spread values (in Hz) were presented in the following three sequences:

*Seq.1*: {62, 96, 152, 241, 479}, {181, 287, 455, 722, 1439}, {227, 359, 569, 902, 1800},

*Seq.2*: {62, 121, 191, 303, 479}, {181, 362, 573, 909, 1439}, {227, 452, 717, 1136, 1800}

*Seq.3*: {62, 76, 191, 381, 479}, {181, 228, 573, 1144, 1439}, {227, 285, 717, 1430, 1800}

##### 3.1.2.3. Spectral Skewness

The Skew-normal distribution (Azzalini, 2005) is a three-parameter family of curves and was employed for constructing stimuli with different skewness while the centroid and spread were being kept constant (Figure 1). The restrictions in the synthesis process that were taken into account with respect to the selection of centroids and spreads were similar to the ones mentioned above, with the additional constraint that skewness in the Skew-normal distribution can only vary within a range of [−0.9953, 0.9953]. Three sound sets were constructed with centroids spaced at 1640, 5600, and 7800 Hz, and spreads at 360, 1,080, and 1,440 Hz, respectively. The spectral skewness values presented in each sequence for a particular centroid were the following:

*Seq.1*: {–0.88, –0.33, 0.00, 0.71, 0.96}

*Seq.2*: {–0.88, –0.11, 0.25, 0.60, 0.96}

*Seq.3*: {–0.88, –0.55, 0.00, 0.87, 0.96}

##### 3.1.2.4. Odd-to-Even Ratio

The stimuli of these sound sets were constructed with 9 harmonics to ensure that roughness would not be a major factor in listeners' ratings. The odd-to-even ratio was controlled by equally attenuating the level in dB of the even harmonics while keeping the odd harmonics fixed at 0 dBFS (dB relative to full scale), and by attenuating the level of the odd harmonics while keeping the even harmonics fixed. In each of those cases, the f0 level was kept fixed at 0 dBFS (Figure 1). Three sound sets with the same attenuation levels were constructed for each of the three f0s at 120, 300, and 720 Hz. The odd-to-even ratio values presented in each sequence for a particular f0 were the following:

*Seq.1*: {0.251, 0.648, 1.25, 3.14, 1250}

*Seq.2*: {0.251, 0.501, 1.25, 4.98, 1250}

*Seq.3*: {0.251, 0.881, 1.25, 1.98, 1250}

##### 3.1.2.5. Spectral Deviation

The reference spectrum was selected from a sample of one thousand amplitude distributions that were generated by randomly choosing the level of each harmonic from a uniform distribution covering the range of [−60, 0] dBFS. The amplitude distribution that had the greatest spectral deviation along with an odd-to-even ratio of approximately 1 and the greatest T2 tristimulus value (relative proportion of energy in harmonics 2-4) (Peeters et al., 2011) below the level of the f0 was chosen as the reference spectrum for constructing stimuli with controlled deviations. The decision to choose an odd-to even ratio of approximately 1 (Table 1) ensured that this sound set did not vary predominantly according to that parameter (which was tested separately), whereas the choice of having the greatest possible T2 ensured that most of the deviation resulted from the differences in level among the upper harmonics. Choosing a T2 below the level of the f0 (i.e., the T1) ensured that the pitch would be the same with f0 and not an octave higher. The spectral deviation was then controlled by reducing the differences in level between the successive harmonics of a reference spectrum until all harmonics had reached a level of 0 dBFS (Figure 1). In total, three sound sets with f0s at 120, 300, and 720 Hz were constructed. For these sound sets, the number of harmonics was increased to 16, which enabled the generation of a more uniform sample of amplitude distributions and the evaluation of a wider range of deviations that occur between the higher harmonics. The spectral deviation values presented in each sequence for a particular f0 were the following (×10^2^):

*Seq.1*: {0, 2.42, 4.76, 5.45, 5.75}

*Seq.2*: {0, 3.40, 5.17, 5.62, 5.75}

*Seq.3*: {0, 1.68, 4.58, 5.52, 5.75}

At this point it should be mentioned that the odd-to-even ratio stimulus set had a greater range of spectral deviation (Table 1) because all the odd or even harmonic components had a minimum level of –60 dBFS, whereas in the spectral deviation sets only one out of the sixteen components had a level at –60 dBFS.

##### 3.1.2.6. Spectral Slope

The spectral slope of each stimulus was controlled by reducing, or increasing, the levels in dB of 9 successive harmonics between the extremes of a flat and 1/*n*^4^ (i.e., 24 dB/octave), or 1/((*N*+1)−*n*)^4^, harmonic amplitude spectra for negative and positive slopes, respectively, where *n* is the harmonic number and *N* is the total number of harmonics (Figure 1). In total, three sound sets with f0s at 120, 300, and 720 Hz were constructed for both positive and negative slopes the values of which were computed using linear regression over the power in dB of log-spaced harmonics. The spectral slope values (in dB/octave) presented in each sequence for a particular f0 were the following:

*Seq.1*: {–24, –12, –1, 2, 5}

*Seq.2*: {–24, –16, –4, 0, 5}

*Seq.3*: {–24, –6, 0, 4, 5}

#### 3.1.3. Procedure

In every trial, listeners were first presented with a sequence of five stimuli that varied (monotonically) along an audio feature. Then, they had to adjust the spacing of five markers presented on screen according to their perception of the relative spacing of the five stimuli in terms of differences between their successive audio feature levels. The first and last markers corresponding to the first and last stimuli were kept fixed. In other words, the separation between the markers reflected how far apart from each other the stimuli were perceived to be: the interval between them. The order of presentation of features and the corresponding f0s or centroids of a given feature were randomized. For each feature, the three different sequences of stimulus values were presented randomly and in both ascending and descending orders. The experiment took approximately 60 min to complete.

The user interface was programmed in PsiExp (Smith, 1995). Sounds were amplified through a Grace Design m904 monitor (Grace Digital Audio, San Diego, CA) and presented diotically over Sennheiser HD600 headphones (Sennheiser Electronic GmbH, Wedemark, Germany). The range of the presentation levels over all stimuli was 53.4–74 dBA as measured with a Brüel & Kjær Type 2205 sound-level meter with a Brüel & Kjær Type 4153 artificial ear to which the headphones were coupled (Brüel & Kjær, Nærum, Denmark). Listeners were seated individually in an IAC model 120act-3 double-walled audiometric booth (IAC Acoustics, Bronx, NY).

### 3.2. Results

The interval scales were constructed by fitting a proper function on the median values of the ratings computed by first averaging each participant's ratings on the ascending and descending sequences of stimulus values in order to control for any hysteresis effects, which occur when the order of presentation affects the judgment of successive intervals between stimuli (Stevens, 1975). The criteria used for choosing the form of the function were monotonicity and maximum explained variance (*R*^2^). The best fitting function in relation to the above criteria was determined after evaluating the performance of exponential, power, and polynomial functions which ranged from linear to the maximum allowable degree. In cases where the best fitting function was a power function, it was necessary to first transpose the feature values of the stimuli to strictly positive by adding an (offset) constant before applying the fitting algorithm. The reliability of listeners' ratings on a particular sequence of feature values was estimated according to Cronbach's *alpha* (α), which was computed on the averaged ratings of each participant between the ascending and descending conditions of each sequence (Stevens, 1975). Cronbach's *alpha* was estimated separately for each sequence rather on the combined ratings of all sequences, because some stimuli were presented in more than one sequence, whereas others were presented in just one. For qualitatively interpreting Cronbach's *alpha*, we used the rule of thumb proposed by George and Mallery (2003): α < 0.5 – unacceptable; 0.5 ≤ α < 0.6 – poor; 0.6 ≤ α < 0.7 – questionable; 0.7 ≤ α < 0.8 – acceptable; 0.8 ≤ α < 0.9 good; α≥0.9 – excellent. The final median and interquartile ranges of listeners' ratings and the fitting functions that are shown in the respective plots were computed on each stimulus value after combining its ratings from all the sequences in which it was presented and after averaging each participant's ratings for the ascending and descending conditions. For cases in which the fitting function had to be applied on transposed stimulus values, the respective plots display the actual stimulus values on the *x*-axis. The *R*^2^ value of the fit of each equation to the mean data is indicated with the regression equation for each descriptor below. All analyses were done in Matlab and R (R Core Team, 2013).

#### 3.2.1. Spectral Centroid

**Figure 2:**
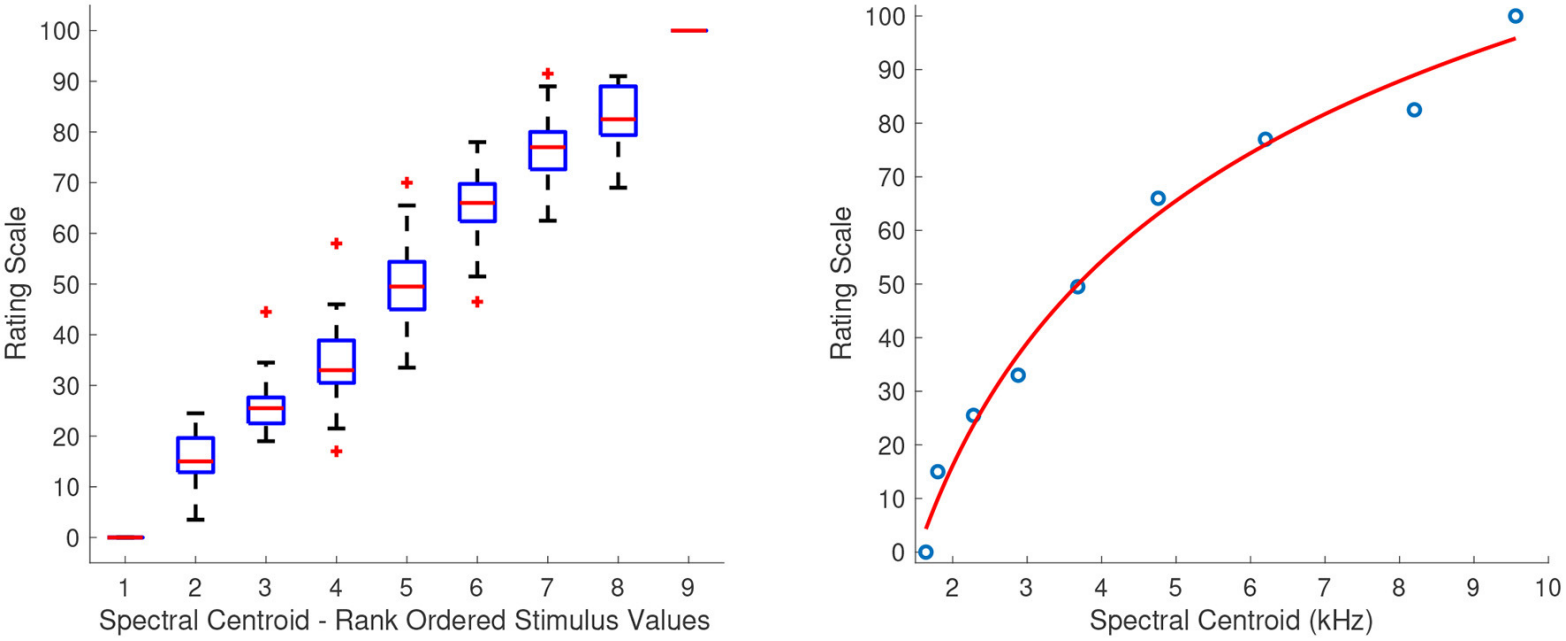
Boxplots (left panel) and shape of the fitting function (right panel) for spectral centroid. Whiskers extend to 2.7 SD.

Figure 2 shows the combined median ratings and interquartile ranges calculated over all stimulus sequences (left panel) and the shape of the fitting function (right panel), from which it can be seen that the ratings increase monotonically for increasing spectral centroid. The second sequence in which the intermediate stimulus values were clustered around the middle one, had a good reliability score (α = 0.82). The first sequence in which the intermediate values were more equidistant had a considerably lower but still acceptable reliability score (α = 0.72). The third sequence in which the intermediate values were clustered around the edges near the first and last stimuli had the lowest score which indicated an unacceptable reliability (α = 0.41). The fitting function was a power function of the form:


(1) $$\begin{array}{l} f\left(x\right)=-1284\cdot x^{-0.1824}+337.2,\;R^{2}=0.99 \end{array}$$

#### 3.2.2. Spectral Spread

**Figure 3:**
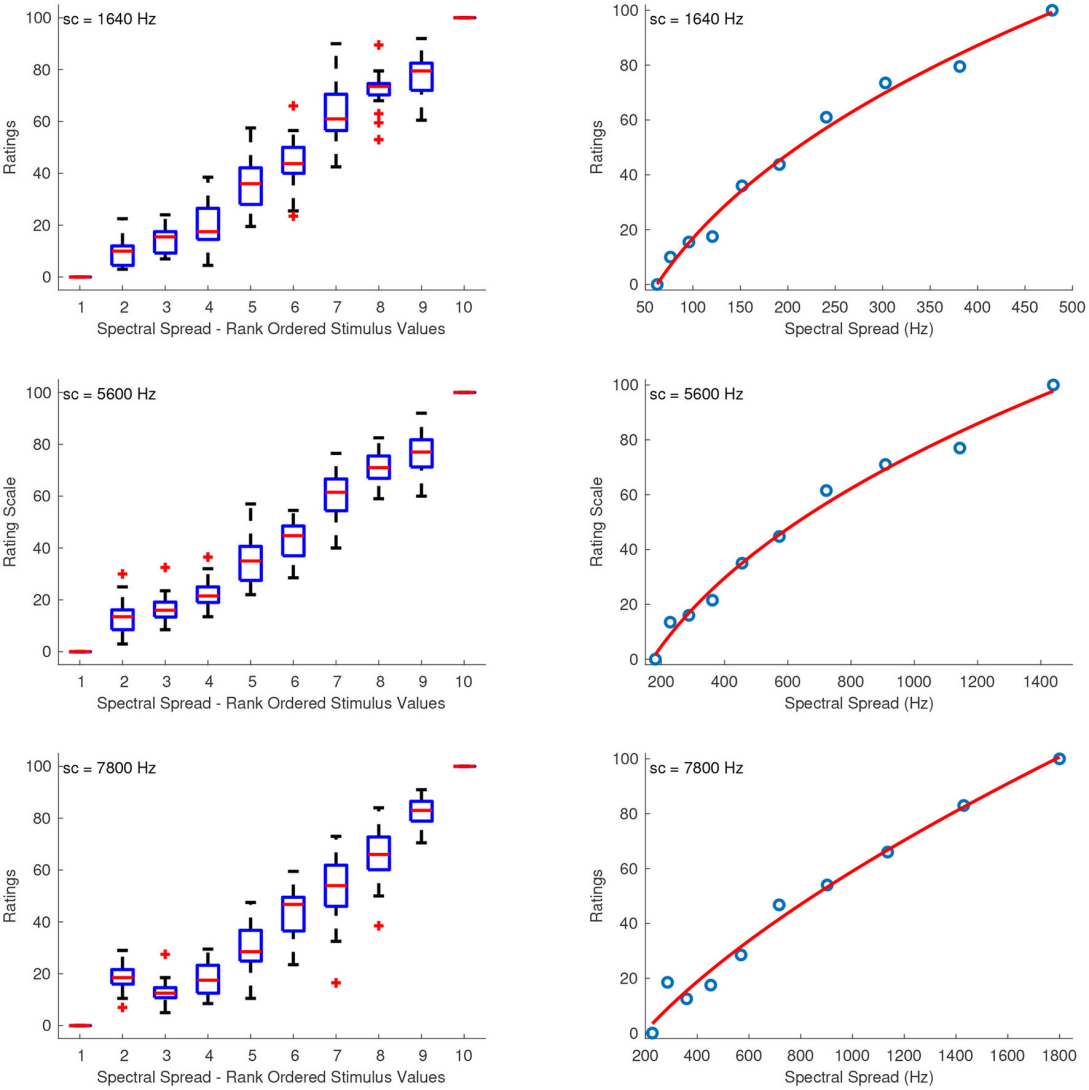
Boxplots (left panels) and shape of the fitting functions (right panels) for spectral spread at three values of spectral centroid (sc). Whiskers extend to 2.7 SD.

Figure 3 shows the combined median ratings and interquartile ranges calculated over all stimulus sequences, and the shape of the fitting function. The median ratings increased monotonically for increasing spectral spread with the exception of the sound set at a centroid of 7,800 Hz, in which the second stimulus in the combined spacing was overestimated by most listeners. Overall, the reliability was good to marginally acceptable for the interval estimations of spectral spread, with α ranging from 0.69 to 0.86. The only exceptions were the third sequences which had their intermediate values clustered around the edges of the first and last stimuli with centroids at 5,600 and 7,800 Hz, and which exhibited questionable to unacceptable reliability with α's at 0.6 and 0.34, respectively. The fitting functions were all power functions which had the following coefficients for sound sets with centroids at 1,640, 5,600, and 7,800 Hz, respectively:


(2) $$\begin{array}{l} f_{1640}\left(x\right)=18.26\cdot x^{0.3756}-86.08,\;R^{2}=0.99 \end{array}$$

(3) $$\begin{array}{l} f_{5600}\left(x\right)=10.02\cdot x^{0.3917}-75.15,\;R^{2}=0.99 \end{array}$$

(4) $$\begin{array}{l} f_{7800}\left(x\right)=1.048\cdot x^{0.6451}-31.25,\;R^{2}=0.99 \end{array}$$

The fact that the last fitting function *f*_7800_(*x*) has considerably different coefficients than the other two, reflects the unequal spacing of spectral spread between the sound sets of different spectral centroids.

#### 3.2.3. Spectral Skewness

**Figure 4:**
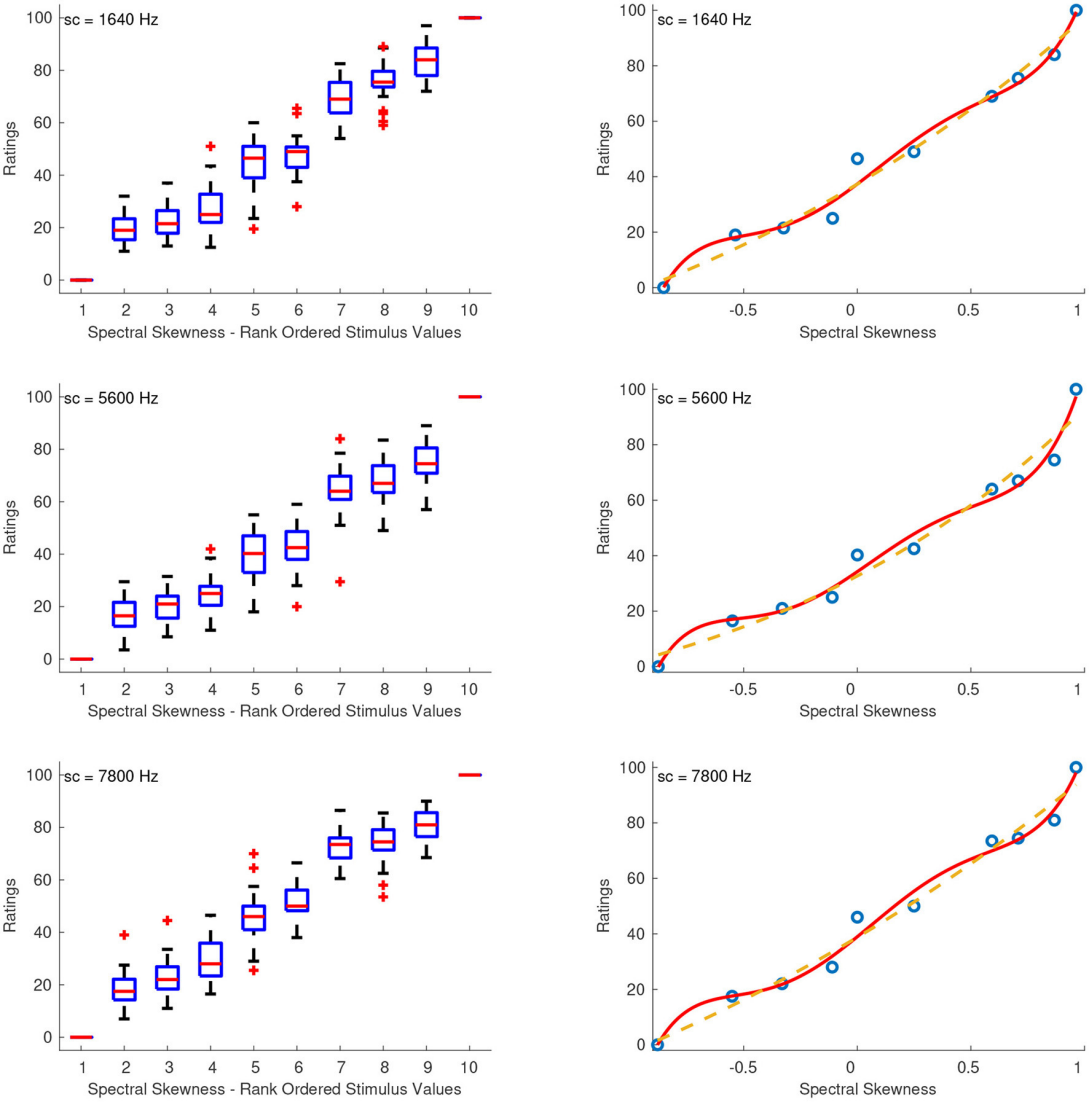
Boxplots (left panels) and shape of the fitting functions (right panels) for spectral skewness at three values of spectral centroid (sc). Whiskers extend to 2.7 SD. Solid line, polynomial function; Dashed line, power function.

Figure 4 shows the combined median ratings and interquartile ranges calculated over all stimulus sequences and the shape of the fitting function. The sequences that had the lowest reliability scores ranging from unacceptable to questionable were the first (α = 0.58) and third (α = 0.46) with centroid at 1640 Hz, as well as the third one (α = 0.68) with centroid at 7,800 Hz. For the rest of the sequences, the reliability ranged from acceptable to good (0.72 ≤ α ≤ 0.80). From the boxplots in the left panel of Figure 4, it can be seen that although the median ratings increase monotonically for increasing spectral skewness, the stimuli seem to be grouped into three different clusters: 2 to 4, 5 to 6, and 7 to 9. This trend of the data was best captured with a fifth order polynomial (solid curves), which had the following coefficients for each sound set with centroids at 1,640, 5,600, and 7,800 Hz, respectively:


(5) $$\begin{array}{l} f_{1640}\left(x\right)=91.21x^{5}-24.86x^{4}-83.3x^{3}+24.59x^{2}+61.63x\\ +37.37,\;R^{2}=0.99 \end{array}$$

(6) $$\begin{array}{l} f_{5600}\left(x\right)=99.72x^{5}-9.505x^{4}-88.99x^{3}+15.49x^{2}+55.97x\\ +34.24,\;R^{2}=0.99 \end{array}$$

(7) $$\begin{array}{l} f_{7800}\left(x\right)=95.34x^{5}-17.78x^{4}-96.95x^{3}+19.02x^{2}+66.96x\\ +38.97,\;R^{2}=0.99 \end{array}$$

Although power functions (dashed curves) provided slightly lower but comparable R-squared values (0.98, 0.96, and 0.98 for centroids at 1,640, 5,600, and 7,800 Hz, respectively) than the fifth-order polynomials, they do not adequately represent the clustered ratings between negative, zero, and positive skewness.

#### 3.2.4. Odd-to-Even Ratio

**Figure 5:**
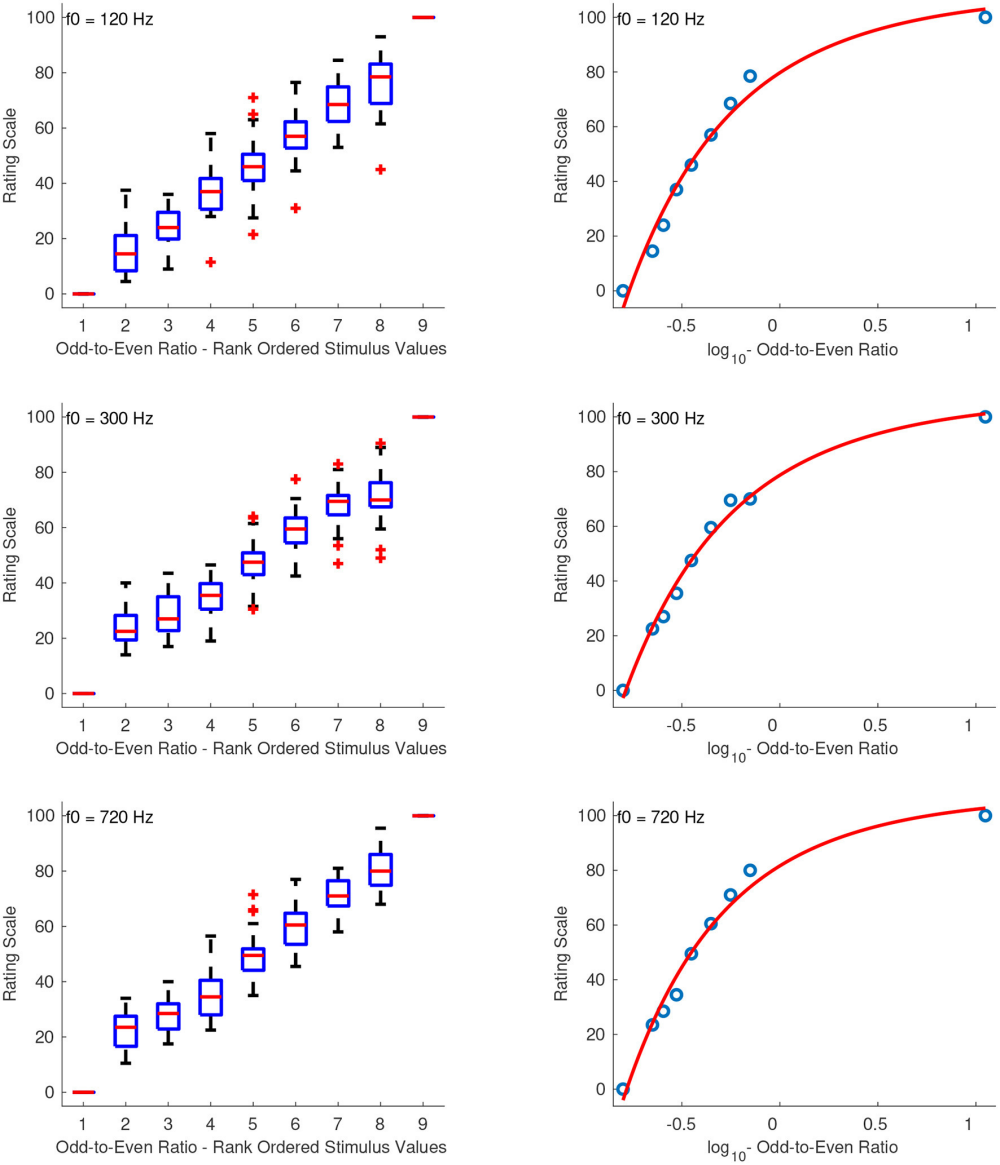
Boxplots (left panels) and shape of the fitting functions (right panels) for odd-to-even ratio at three f0s. Whiskers extend to 2.7 SD.

Figure 5 shows the combined median ratings and interquartile ranges calculated over all stimulus sequences and the shape of the fitting function. From the boxplots it can be seen that the median ratings increased monotonically with increasing odd-to-even ratio. The reliability of interval estimations ranged from poor to excellent (0.53 ≤ α ≤ 0.89). The first sequence had a questionable reliability score with α's at 0.64, 0.63, and 0.66, for f0s at 120, 300, and 720 Hz, respectively. The second sequence had α's at 0.53, 0.77, and 0.56, and the third sequence had α's at 0.89, 0.56, and 0.67, for f0s at 120, 300, and 720 Hz, respectively.

Because of the large range of odd-to-even ratios, the values were first *log*_10_-transformed. All of the stimulus sets were best fit with a power function and were therefore transposed to strictly positive before fitting the function according to: $x^{\prime }=log_{10}\left(x\right)+10$, where *x* is the original stimulus value. The constant value of +10 ensured that the scale includes ratio values greater than $log_{10}\left(10^{-10}\right)$. The following coefficients were used for each sound set with f0s at 120, 300, and 720 Hz, respectively:


(8) $$\begin{array}{l} f_{120}\left(x^{\prime }\right)=\left(-2.904\cdot 10^{10}\right){x\prime }^{-8.63}-109.6,\;R^{2}=0.98 \end{array}$$

(9) $$\begin{array}{l} f_{300}\left(x^{\prime }\right)=\left(-2.061\cdot 10^{10}\right){x\prime }^{-8.499}+107.7,\;R^{2}=0.99 \end{array}$$

(10) $$\begin{array}{l} f_{720}\left(x^{\prime }\right)=\left(-9.95\cdot 10^{10}\right){x\prime }^{-9.196}+108,\;R^{2}=0.98 \end{array}$$

#### 3.2.5. Spectral Deviation

**Figure 6:**
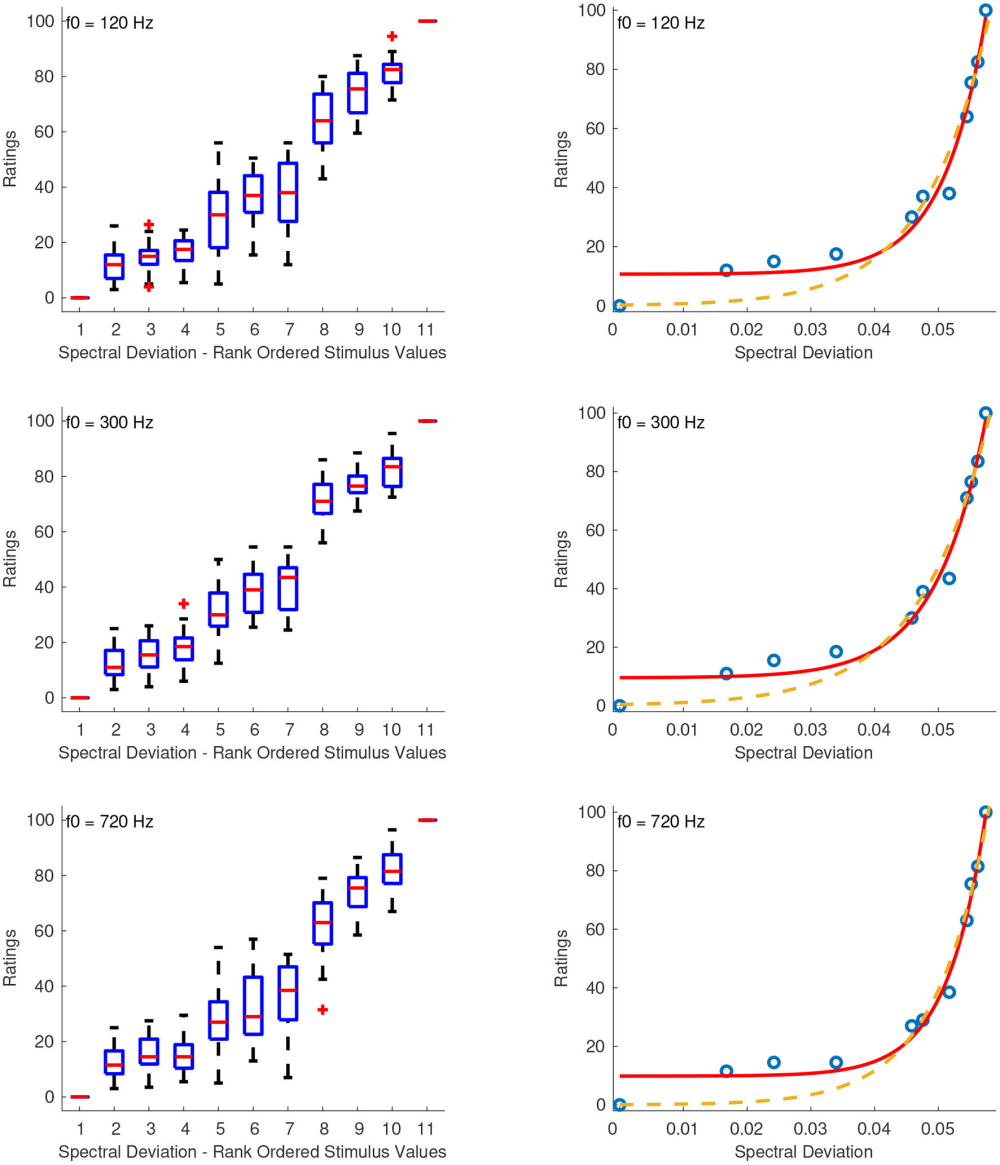
Boxplots (left panels) and shape of the fitting functions (right panels) for spectral deviation at three f0s. Whiskers extend to 2.7 SD. Solid line, power function with the additive term; Dashed line, power function without the additive term.

Figure 6 shows the combined median ratings and interquartile ranges calculated over all stimulus sequences and the shape of the fitting function plotted on the actual stimulus values of spectral deviation. With the exception of the stimulus set at the f0 of 720 Hz, in which the third stimulus was ranked higher than its neighboring stimulus values, the rest of the median values in all sound sets increased monotonically with increasing spectral deviation. The highest reliability scores were good and were observed for the second sequence in which the intermediate stimuli were spaced closer to the middle stimulus value and had α's at 0.83, 0.80, and 0.86 for f0s at 120, 300, and 720 Hz, respectively. The reliability scores for the first and third sequences were 0.72, 0.70, 0.76, and 0.56, 0.76, 0.08, for the three f0s, respectively. For all sound sets, the best fitting function was a power function and, as in the previous case, the values were transposed to strictly positive according to: *x*′ = *x* + 1, where *x* is the original stimulus value and 1 is an arbitrary constant. The following coefficients were used for each sound set with f0s at 120, 300, and 720 Hz, respectively:


(11) $$\begin{array}{l} f_{120}\left(x^{\prime }\right)=\left(14.22\cdot 10^{-3}\right){x\prime }^{156.1}+10.7,\;R^{2}=0.98 \end{array}$$

(12) $$\begin{array}{l} f_{300}\left(x^{\prime }\right)=\left(47.76\cdot 10^{-3}\right){x\prime }^{134.6}+9.563,\;R^{2}=0.98 \end{array}$$

(13) $$\begin{array}{l} f_{720}\left(x^{\prime }\right)=\left(55.78\cdot 10^{-4}\right){x\prime }^{173.3}+9.844,\;R^{2}=0.98 \end{array}$$

Nonetheless, the shapes of the above functions (Figure 6) pose a theoretical problem as the estimated value of the first (anchor) stimulus presented in the series is higher than its theoretical value. This could imply that interval estimations were not based on the anchor stimulus but rather on the second one within each series. Although this hypothesis is supported from the anomalous point shown in the 720 Hz stimulus set, it is not the case for the ratings of the other two sets. This problem can be elevated by fitting a power function without the additive term at the expense of a slightly lower R-squared value while providing a more realistic picture of the marginal supraliminal stimuli (i.e., the first four stimuli) of each stimulus set (Figure 6):


(14) $$\begin{array}{l} f_{120}\left(x^{\prime }\right)=\left(25.48\cdot 10^{-2}\right){x\prime }^{105.6},\;R^{2}=0.94 \end{array}$$

(15) $$\begin{array}{l} f_{300}\left(x^{\prime }\right)=\left(42.69\cdot 10^{-2}\right){x\prime }^{96.53},\;R^{2}=0.96 \end{array}$$

(16) $$\begin{array}{l} f_{720}\left(x^{\prime }\right)=\left(83.83\cdot 10^{-3}\right){x\prime }^{126},\;R^{2}=0.95 \end{array}$$

#### 3.2.6. Spectral Slope

**Figure 7:**
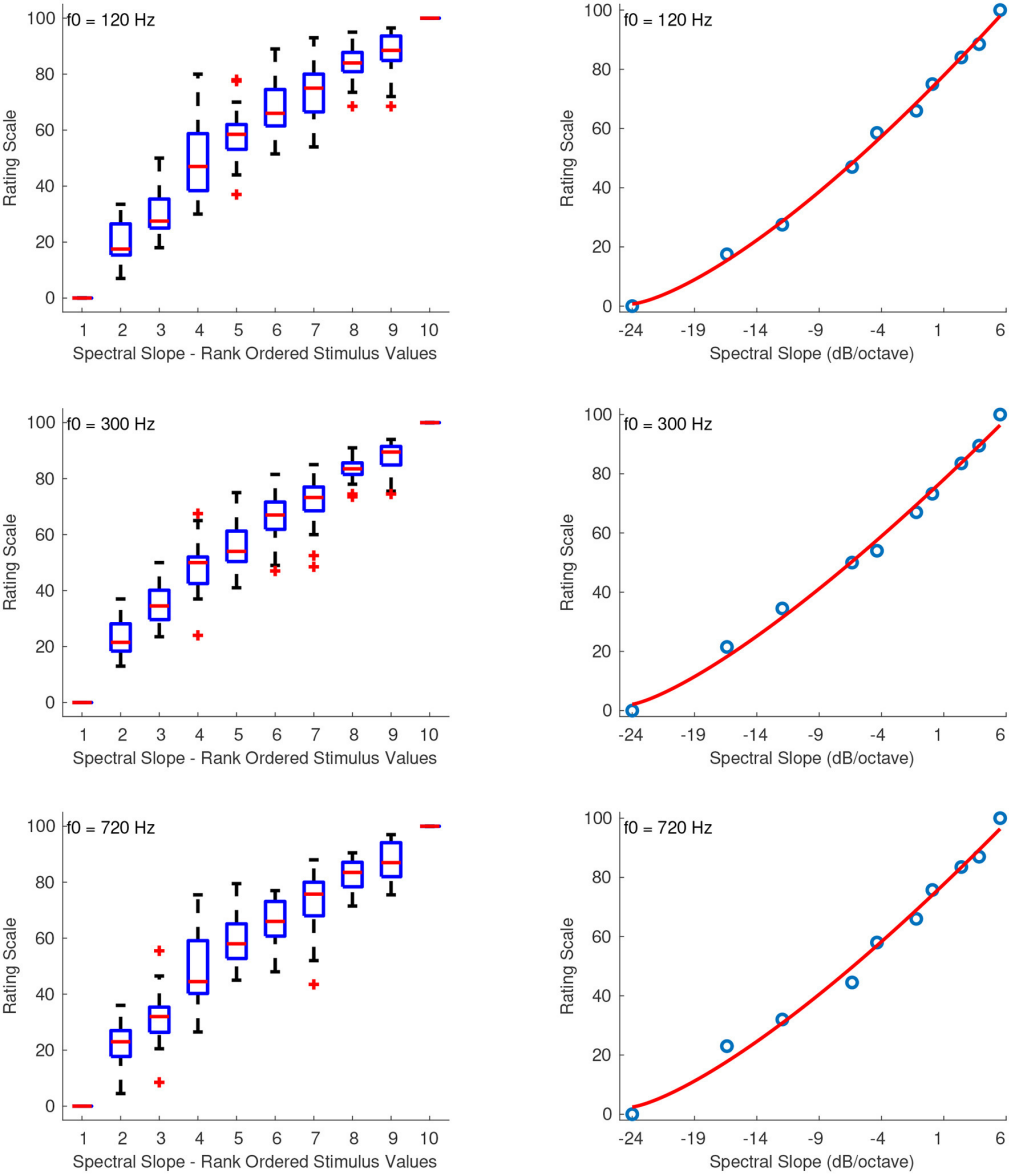
Boxplots (left panels) and shape of the fitting functions (right panels) for spectral slope at three f0s. Whiskers extend to 2.7 SD.

Figure 7 shows the combined median ratings and interquartile ranges calculated over all stimulus sequences and the shape of the fitting function plotted on the actual stimulus values of spectral slope. In all sound sets, the median values increased monotonically with increasing spectral slope. As in the previous feature set of spectral deviation, the highest reliability scores were observed for the third sequence of this sound set in which the intermediate stimuli were spaced closer to the middle stimulus value. For that sequence, the reliability scores were overall marginally excellent with α's at 0.92, 0.89, and 0.89 for f0s at 120, 300, and 720 Hz, respectively. The first and second sequences had overall lower reliability with α's at 0.85, 0.71, 0.75 and 0.64, 0.71, 0.64 for the three f0s, respectively. The second sequence in which the intermediate stimulus values ranged from negative to zero spectral slope seems to have had an effect on the reliability of stimulus ratings, which were overall questionable. The fitting function was again a power function, and, as in the previous cases, the values had to be rescaled to strictly positive before applying the fitting function according to: *x*′ = *x* + 24, where *x* is the original stimulus value. The rescaling constant of +24 allowed values above –24 dB/octave to be included in the scale. The coefficients of the power function for sound sets with f0s at 120, 300, and 720 Hz, are the following:


(17) $$\begin{array}{l} f_{120}\left(x^{\prime }\right)=0.8718{x\prime }^{1.393}+0.7432,\;R^{2}=1 \end{array}$$

(18) $$\begin{array}{l} f_{300}\left(x^{\prime }\right)=1.144{x\prime }^{1.303}+2.16,\;R^{2}=1 \end{array}$$

(19) $$\begin{array}{l} f_{720}\left(x^{\prime }\right)=1.02{x\prime }^{1.336}+2.46,\;R^{2}=0.99 \end{array}$$

### 3.3. Discussion

With the exception of the two anomalous points in one of the sound sets of spectral spread and spectral deviation, the rest of the median values of the interval estimations increased monotonically with increasing stimulus values, which indicates that the experiment was successful and that the listeners were able to estimate intervals of the tested audio features. However, it is well-known that one of the biases of interval scaling usually results from the initial selection and the limited pool of stimuli used for the estimations. This bias was controlled for by presenting subsets of an initial pool of stimuli covering a wide range of each feature with different spacing in each trial. Furthermore, any hysteresis effects were taken into account by presenting the stimulus sequences in both ascending and descending directions. Although we tried to control for the aforementioned biases, another important source of bias that was not possible to account for was the centering tendency, which afflicts all rating scales (Stevens, 1975). As became evident from most of the plots of the fitting functions, listeners tended to use the more central positions of the rating scale and avoid the extremes. The forms of the derived fitting functions were the same within each audio feature when tested at different ranges albeit with different coefficients which indicates that the listeners' perceptions of these features depend on the spectral location at which each feature is presented in terms either of fundamental frequency or of spectral centroid.

The large variability of the reliability scores measured according to Cronbach's *alpha* for each set of stimuli within the same feature and for a particular sequence indicates that the spacing of the stimuli had a big effect on the internal consistency of the listeners. The lowest alphas were observed for the stimuli of spectral skewness with centroid at 1,640 Hz and for the odd-to-even ratio. In addition, the overall lowest reliabilities were mainly observed for the sequences in which the stimuli were not (approximately) equidistantly spaced, and when the second and next-to-last stimuli were placed closer to the edges rather than to the middle stimulus value of the sequence. We hypothesize that this could be because listeners were using the middle stimulus of the sequence as a reference (standard stimulus) for their interval estimations and as previously mentioned, judgments tend to be more accurate for stimuli placed closer to the standard rather than far away from it (Gescheider and Hughson, 1991). Another factor that might have played a role in the observed variance of interval estimations, the anomaly points, and for some cases in the relatively low reliability scores, could be that for some features, the audible differences between the stimuli in the combined set were marginally supraliminal (albeit clearly supraliminal within each sequence). However, this was a direct consequence of the narrow perceivable range of some features (e.g., odd-to-even ratio) and the constraints imposed by the synthesis procedure for constructing the stimuli (e.g., narrow permissible range of spectral skewness due to the Skew-normal distribution).

In conclusion, the largest biases of the derived interval scales resulted from the centering tendency of the listeners and in some cases from the marginally supraliminal spacing of stimulus values. Despite these biases, the experiment should be considered as an exploratory step, which confirmed the ability of the listeners to estimate intervals of the tested audio features. It also allowed us to proceed to the construction of ratio scales presented in the next section.

## 4. Experiment 2: Equisection Scaling

The aim of this experiment was the derivation of ratio scales of audio features provided that listeners are able to estimate intervals, which was confirmed from the results of Experiment 1. In this experiment, listeners had control over the stimulus values and were asked to equisect a continuum of a particular audio feature. Each equisection was performed using the progressive solution (Gescheider, 1997) according to which listeners progressively partition the continuum formed by the stimuli into a number of equal-sounding intervals. The equality of sensory intervals implies that the intervals themselves have ratio properties (Marks and Gescheider, 2002) and thus, the results of this experiment led to ratio scale measurements.

### 4.1. Method

#### 4.1.1. Participants

Twenty participants, 6 female and 14 male, with a median age of 25 years (range: 18–41) were recruited from the Schulich School of Music, McGill University. All of them were self-reported amateur or professional musicians with formal training in various disciplines such as performance, composition, music theory, and sound engineering. Participants were compensated for their time. One participant reported perfect pitch and another one synesthesia.

#### 4.1.2. Stimuli and Presentation

All the stimulus sets were constructed with the procedures described in Section 3.1.2 and at the same f0s and spectral centroids. In order to create a continuum within a range of a particular feature, several stimuli were constructed with multiple imperceptible successive differences. The total number of sounds used for each stimulus set and the ranges of feature values for a particular set are indicated in Table 1. The extreme feature values of each stimulus set were the same as those used in Experiment 1. Figure 1 shows for a particular stimulus set, the spectral envelopes of the anchor and mid-point stimuli used in the rating scales of the present experiment.

#### 4.1.3. Procedure

In a first step, listeners divided the continuum of an audio feature into two equal-sounding intervals, by triggering each stimulus with a cursor along a horizontal bar that contained the stimuli, and by placing a marker over the stimulus-bar. Each section was then bisected in the next step. In total there were three bisections: the first one was made between the stimuli of the total range, and the other two within each of the lower and upper bisected ranges. The order of presentation of the upper and lower half bisections was randomized. In a final step, listeners were presented with all their bisections and were instructed to make further fine adjustments so that all four intervals they had created in the previous steps sounded equal. The order of presentation of features and the f0 or spectral centroid of each feature were randomized. As in the previous experiment, the stimuli were presented in both ascending and descending conditions at separate trials: i.e., the stimulus bar would start either from the lowest (ascending condition) or the highest (descending condition) stimulus value of the feature set. The experiment took approximately 60 min to complete.

### 4.2. Results

The ratio scales were constructed by fitting a function to the median values of each equisection computed on both the ascending and descending presentations of the stimulus sets. As in the previous experiment, whenever the best fitting function was a power function, it was necessary to first transpose the stimulus values to strictly positive before fitting the function. The criteria used for choosing the form of the function were again monotonicity and maximum explained variance, but also good continuation of monotonicity (no oscillations) outside the tested range. After identifying the form of the function, the *zero point* of the scale was determined either empirically by extrapolating the function to a point that marks the lower limit of perception for a particular audio feature or, wherever applicable, according to the physical stimulus value for which “zero” has a physical meaning (e.g., zero skewness). Finally, the units of the psychophysical scales were defined by assigning specific numerals to the points of the equisection scale. Cronbach's *alpha* was again used to evaluate the reliability of the derived scales across listeners. In all cases, the reliability was overall excellent, with the scales of spectral centroid and spectral skewness with centroid at 7,800 Hz having the highest reliability (α = 0.96). The lowest reliability was observed for the equisections of spectral spread with centroid at 7,800 Hz (α = 0.89) and odd-to-even ratio with f0 at 720 Hz (α = 0.78).

#### 4.2.1. Spectral Centroid

**Figure 8:**
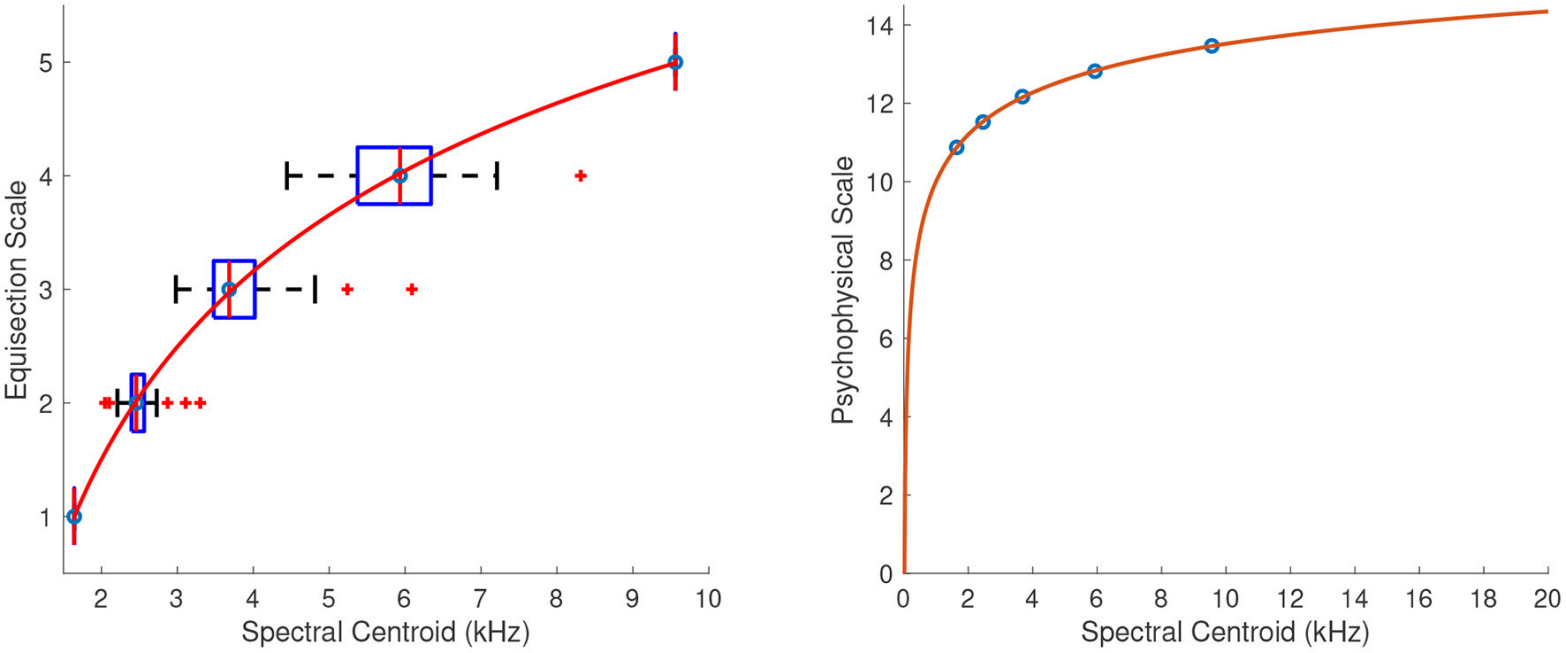
Equisection and psychophysical scales of spectral centroid. On the left: boxplots and fitting function on the median ratings. Whiskers extend to 2.7 SD. On the right: psychophysical scale and extrapolated fitting function.

The left panel in Figure 8 shows the fitted power function on top of the median ratings and interquartile ranges. At that point, the ordinate was assigned arbitrary units which represent equal spectral centroid distances as perceived by listeners. The right panel in Figure 8 shows the extrapolated function for centroids in the range of 20 Hz to 20 kHz. The location of the zero point on the ordinate was assigned the value of 20 Hz, which marks the lowest limit of pitch perception, and finally, the units of the scale were derived by assigning a value of 10 to the 1-kHz spectral centroid. The coefficients of the final fitting equation after the unit assignment are:


(20) $$\begin{array}{l} f\left(x\right)=-34.61x^{-0.1621}+21.2985,\;R^{2}=1 \end{array}$$

#### 4.2.2. Spectral Spread

**Figure 9:**
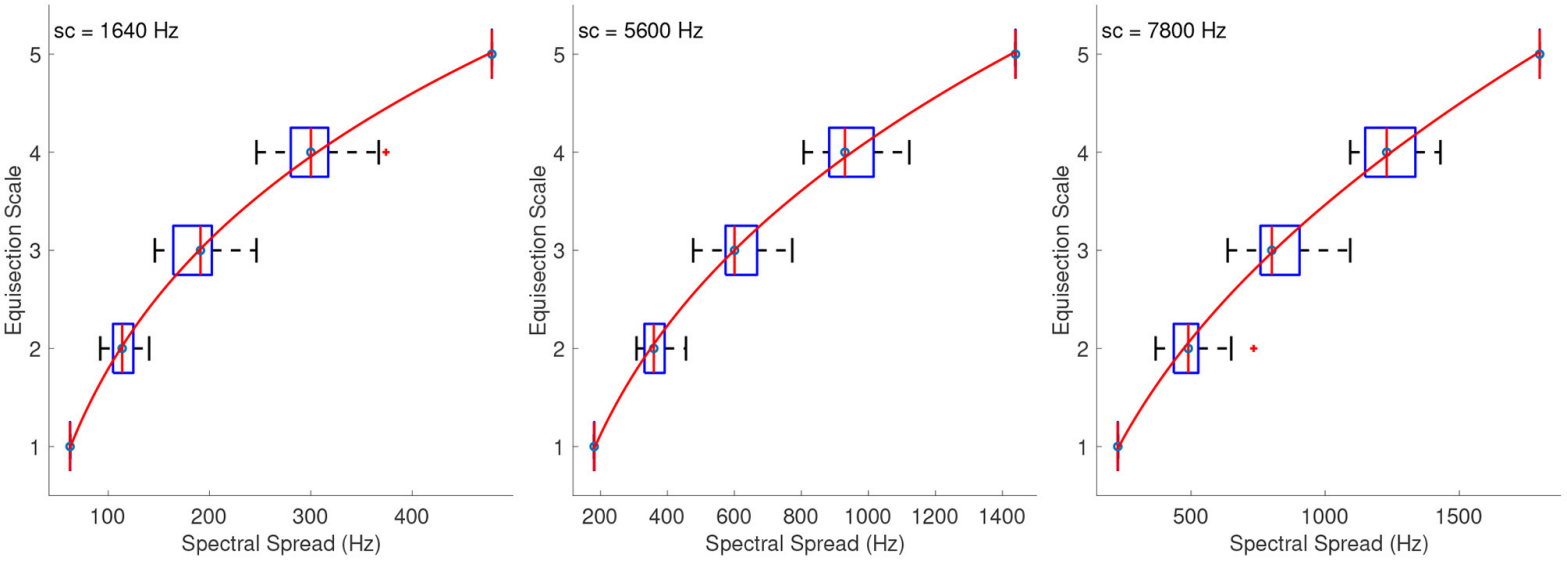
Equisection scales of spectral spread at three values of spectra centroid (sc). Boxplots and fitting function on the median ratings. Whiskers extend to 2.7 SD.

**Figure 10:**
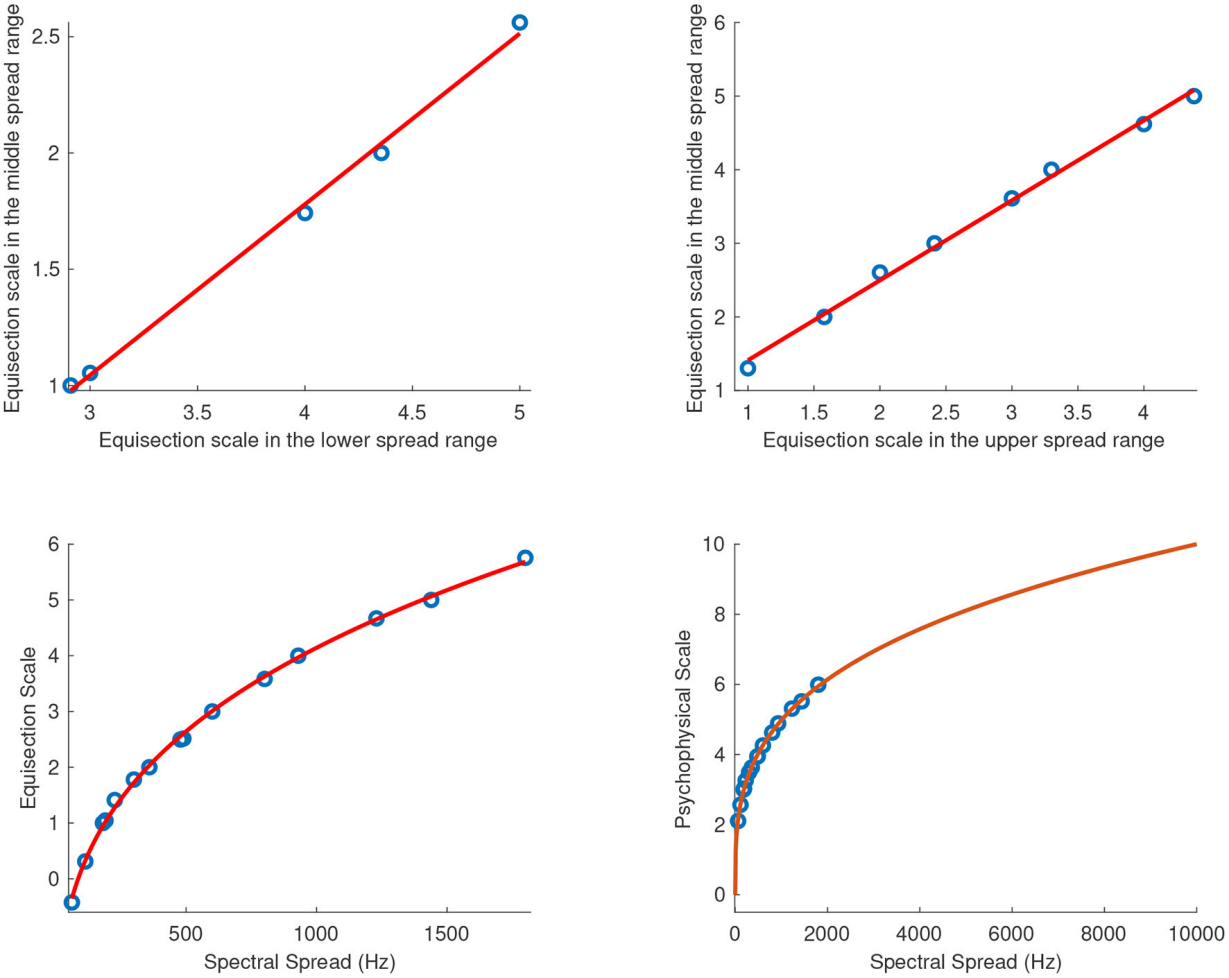
Combined equisection and psychophysical scales of spectral spread. Top panels, equisection scales for lower and upper spread range as a function of middle-spread range; Bottom panels, unified equisection scale (on the left) and psychophysical scale (on the right).

Figure 9 shows the fitted power functions on top of the median ratings and interquartile ranges for the sound sets of spectral spread with centroids at 1,640 (*R*^2^ = 1), 5,600 (*R*^2^ = 1), and 7,800 Hz (*R*^2^ = 1), respectively. In order to find a single psychophysical function of spectral spread covering the entire range independently of the centroid used in each sound set, the three functions for the overlapping tested ranges needed to be combined. To this end, Torgerson's (1958) method was used according to which the scale values in the lower and upper ranges are converted into scale units of the middle range, resulting in a single function. The conversion was performed for the overlapping portions of spectral spread's range by linearly regressing both the lower (*R*^2^ = 1) and upper ranges (*R*^2^ = 1) over the mid-range. The conversion equations for the lower and upper ranges were *f*_*l*_(*x*) = 0.7345*x* − 1.159, and *f*_*u*_(*x*) = 1.086*x* + 0.3256 respectively, and their respective plots are shown in the top panel of Figure 10. The final fitting power function (*R*^2^ = 1) covering the entire tested range is shown on the left of the bottom panel of Figure 10 where the vertical distances on the graph represent spectral-spread distances as perceived by the listeners.

The bottom right panel of Figure 10 shows the extrapolated power function in the range of 0 Hz to 10 kHz^1^. The zero point on the ordinate was assigned the value of 0 Hz because in this case, a spectral spread of 0 Hz has a physical meaning indicating the presence of just a single component in the spectrum. The units of the final scale were derived after assigning a value of 10 to the spread of 10 kHz. The final fitting equation covering the entire tested range and after the unit assignment is:


(21) $$\begin{array}{l} f\left(x\right)=0.6134x^{0.3031},\;R^{2}=1 \end{array}$$

#### 4.2.3. Spectral Skewness

**Figure 11:**
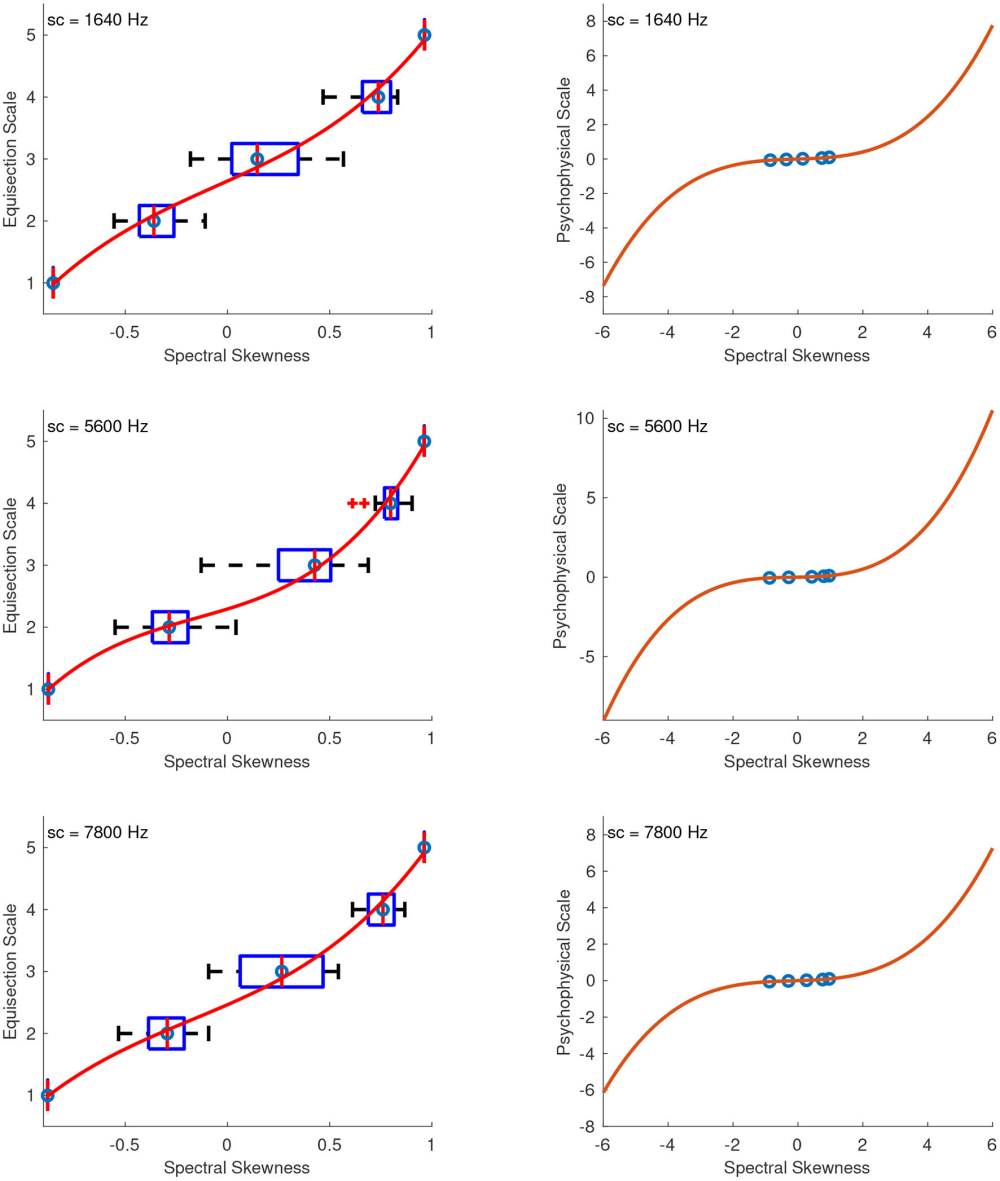
Equisection and psychophysical scales of spectral skewness. Left panels, boxplots and fitting function on the median ratings at three values of spectral centroid (sc). Whiskers extend to 2.7 SD; Right panels, psychophysical scale and extrapolated fitting functions.

Figure 11 shows the fitted third-order polynomial functions on top of the median ratings and interquartile ranges for the sound sets of spectral skewness with centroids at 1,640, 5,600, and 7,800 Hz. The skewness values were the same in all sound sets, so the aim was not to derive a single function independent of the centroid used, as in the previous case, but to derive psychophysical functions of spectral skewness centered at different locations in the spectrum. Figure 11 on the right shows the extrapolated functions in the range of −6 to 6 spectral skewness. As in the previous case, a value of 0 skewness has a physical meaning indicating a gaussian spectral distribution and therefore, the zero point on the ordinate was assigned the value of 0 skewness. Finally, the units of the scale were derived after assigning a value of 0.1 to the skewness of 1. The fitting equations after the unit assignment for skewness at centroids of 1,640, 5,600, and 7,800 Hz are:


(22) $$\begin{array}{l} f_{1640\left(x\right)}=0.03334x^{3}+0.005587x^{2}+0.06107x,\;R^{2}=0.99 \end{array}$$

(23) $$\begin{array}{l} f_{5600\left(x\right)}=0.04435x^{3}+0.02014x^{2}+0.03551x,\;R^{2}=1 \end{array}$$

(24) $$\begin{array}{l} f_{7800\left(x\right)}=0.02951x^{3}+0.01567x^{2}+0.05482x,\;R^{2}=1 \end{array}$$

#### 4.2.4. Odd-to-Even Ratio

**Figure 12:**
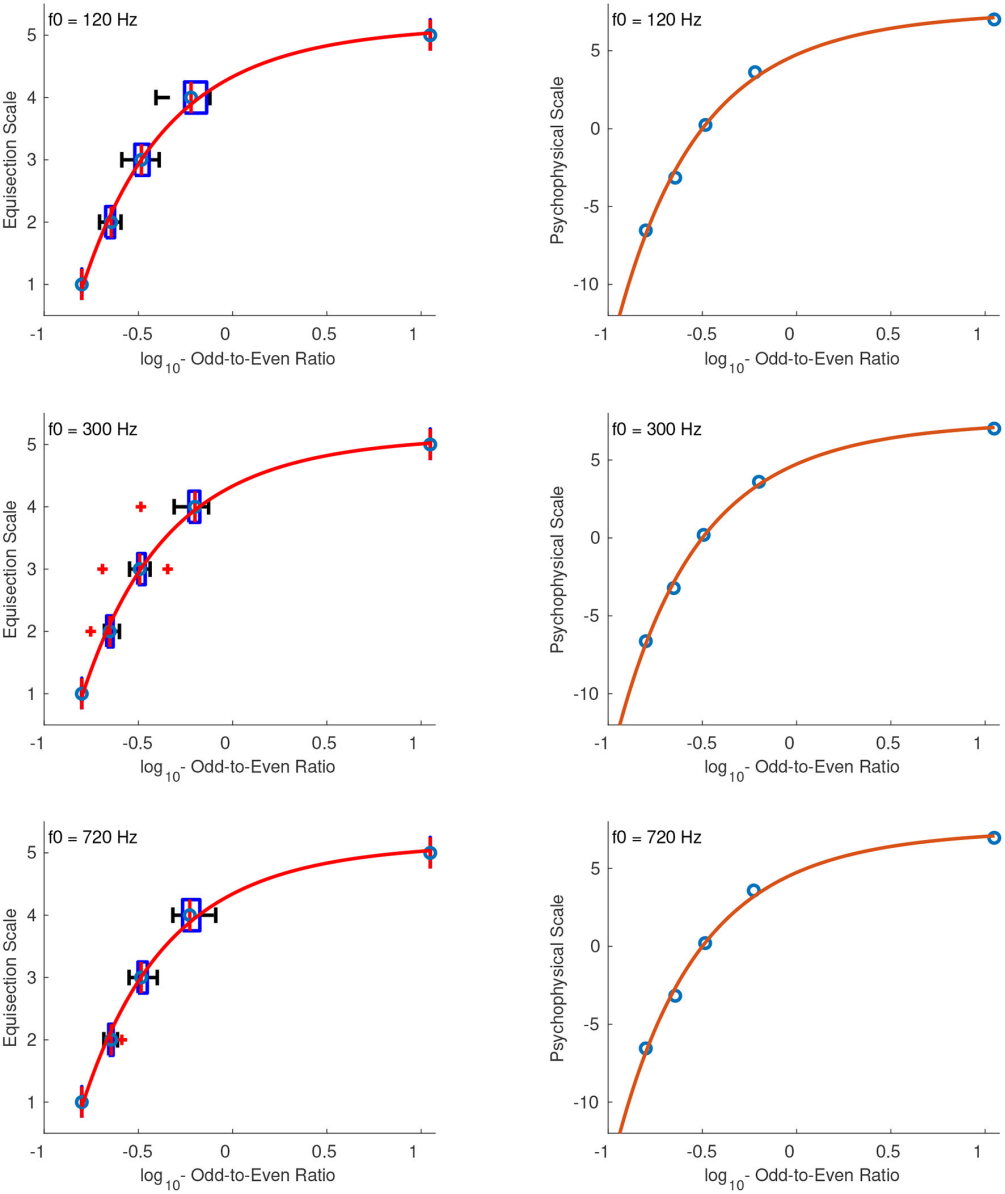
Equisection and psychophysical scales of odd-to-even ratio at three f0s. Left panel, Boxplots and fitting function on the median ratings. Whiskers extend to 2.7 SD; Right panel, psychophysical scale and extrapolated fitting function on *log*_10_-transformed stimulus values.

The best fitting function for the odd-to-even ratio was a power function. Because of the large range of stimulus values *x*, these were first log10-transformed and transposed to strictly positive before fitting the power function according to: $x^{\prime }=log_{10}\left(x\right)+10$. Figure 12 **(left panel)** shows the fitted functions on top of the median ratings and interquartile ranges for the sound sets with f0s at 120, 300, and 720 Hz. The abscissa corresponds to log-transformed values of the odd-to-even ratio, and the arbitrary units on the ordinate represent equal odd-to-even ratio distances as perceived by the listeners. Because the perception of this feature depends on fundamental frequency (Kazazis et al., 2021), the aim was not to find a single function independent of fundamental frequency but to derive more precise psychophysical functions of odd-to-even ratio at the different f0s tested.

The right panel of Figure 12 shows the extrapolated functions in the range of –0.9 to 3 of the *log*_10_-transformed odd-to-even ratios. The zero point of the scale was defined at the odd-to-even ratio of 1, and since the values were log-transformed this point corresponds to the zeros on the abscissas in the right panel of Figure 12. The units of the ordinate were derived after assigning a value of 2 to the odd-to-even ratio of 2. The fitting equations after the unit assignment for the stimuli at f0s of 120, 300, and 720 Hz are:


(25) $$\begin{array}{l} f_{120}\left(x^{\prime }\right)=-1.49\cdot 10^{11}{x\prime }^{-10.29}+7.60,\;R^{2}=1 \end{array}$$

(26) $$\begin{array}{l} f_{300}\left(x^{\prime }\right)=-1.798\cdot 10^{11}{x\prime }^{-10.38}+7.55,\;R^{2}=1 \end{array}$$

(27) $$\begin{array}{l} f_{720}\left(x^{\prime }\right)=-1.808\cdot 10^{11}{x\prime }^{-10.38}+7.55,\;R^{2}=1 \end{array}$$

#### 4.2.5. Spectral Deviation

**Figure 13:**
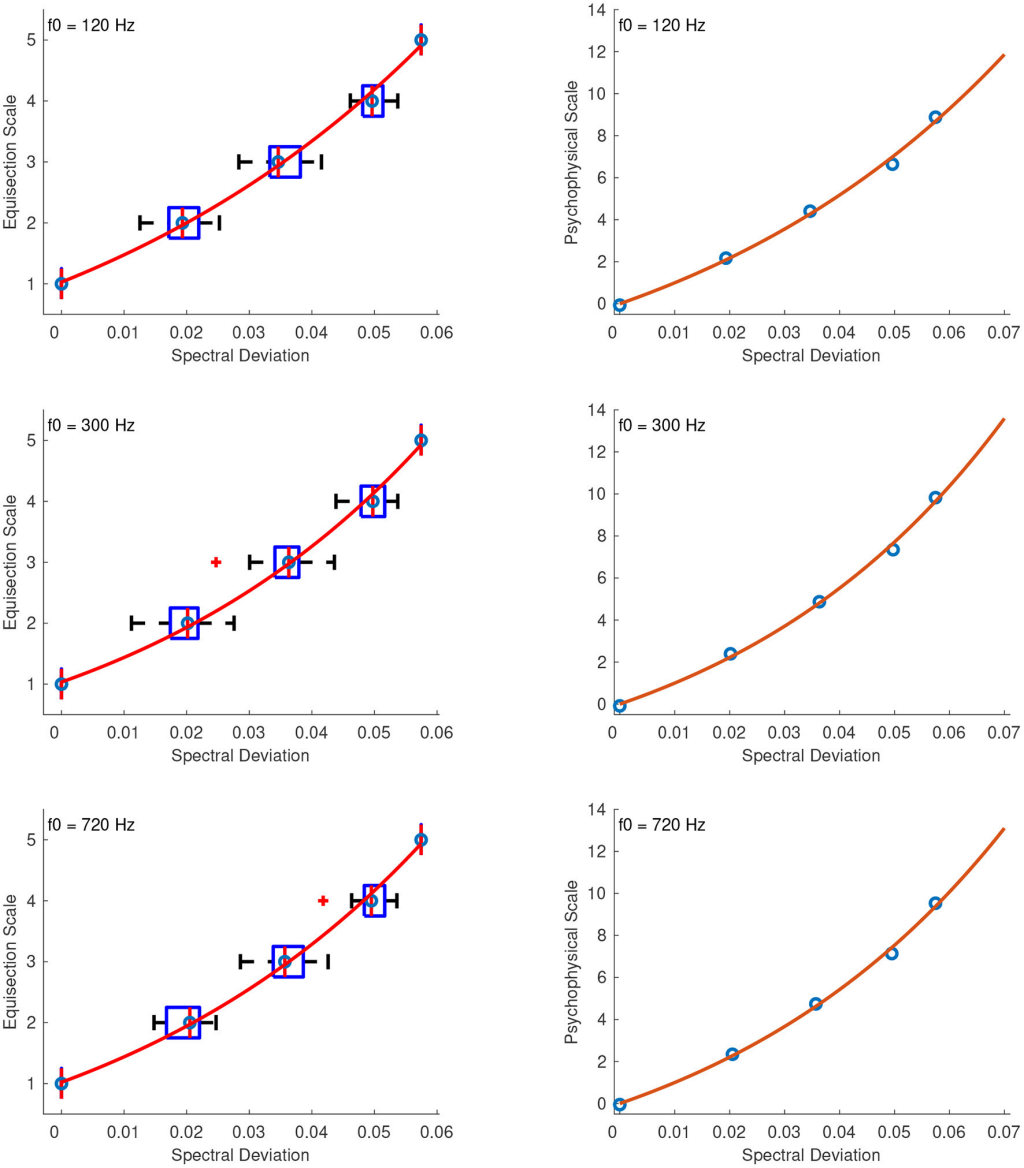
Equisection and psychophysical scales of spectral deviation at three f0s. Left panel, boxplots and fitting function on the median ratings. Whiskers extend to 2.7 SD; Right panel, psychophysical scale and extrapolated fitting function.

The best fitting function for the spectral deviation stimuli was a power function, and, as in the previous case, the stimulus values *x* were transposed to strictly positive before fitting the function according to *x*′ = *x* + 1. Although it would have been possible to perform the fitting after adding a small constant just to the stimulus of zero spectral deviation, the numerical accuracy of the algorithm, and thus the fit, was found to be poorer when compared to shifting all the values by a larger constant, possibly due to round-off errors. Figure 13 **(left panel)** shows the fitted functions on top of the median ratings and interquartile ranges for the stimuli with f0s at 120, 300, and 720 Hz, where the abscissas correspond to the actual stimulus parameter values.

The right panel of Figure 13 shows the fitted functions in the extrapolated range of 0 to 0.07 spectral deviation after the unit assignment. The zero point of the scale was naturally assigned the value of 0, which maps to zero spectral deviation, and finally, the units on the ordinate were derived after assigning a value of 1 to the 0.01 spectral deviation. The final fitting equations for the stimuli at 120, 300, and 720 Hz are, respectively:


(28) $$\begin{array}{l} f_{120}\left(x^{\prime }\right)=5.302{x\prime }^{17.37}-5.30,\;R^{2}=1 \end{array}$$

(29) $$\begin{array}{l} f_{300}\left(x^{\prime }\right)=4.267{x\prime }^{21.16}-4.27,\;R^{2}=1 \end{array}$$

(30) $$\begin{array}{l} f_{720}\left(x^{\prime }\right)=4.504{x\prime }^{20.15}-4.50,\;R^{2}=1 \end{array}$$

#### 4.2.6. Spectral Slope

**Figure 14:**
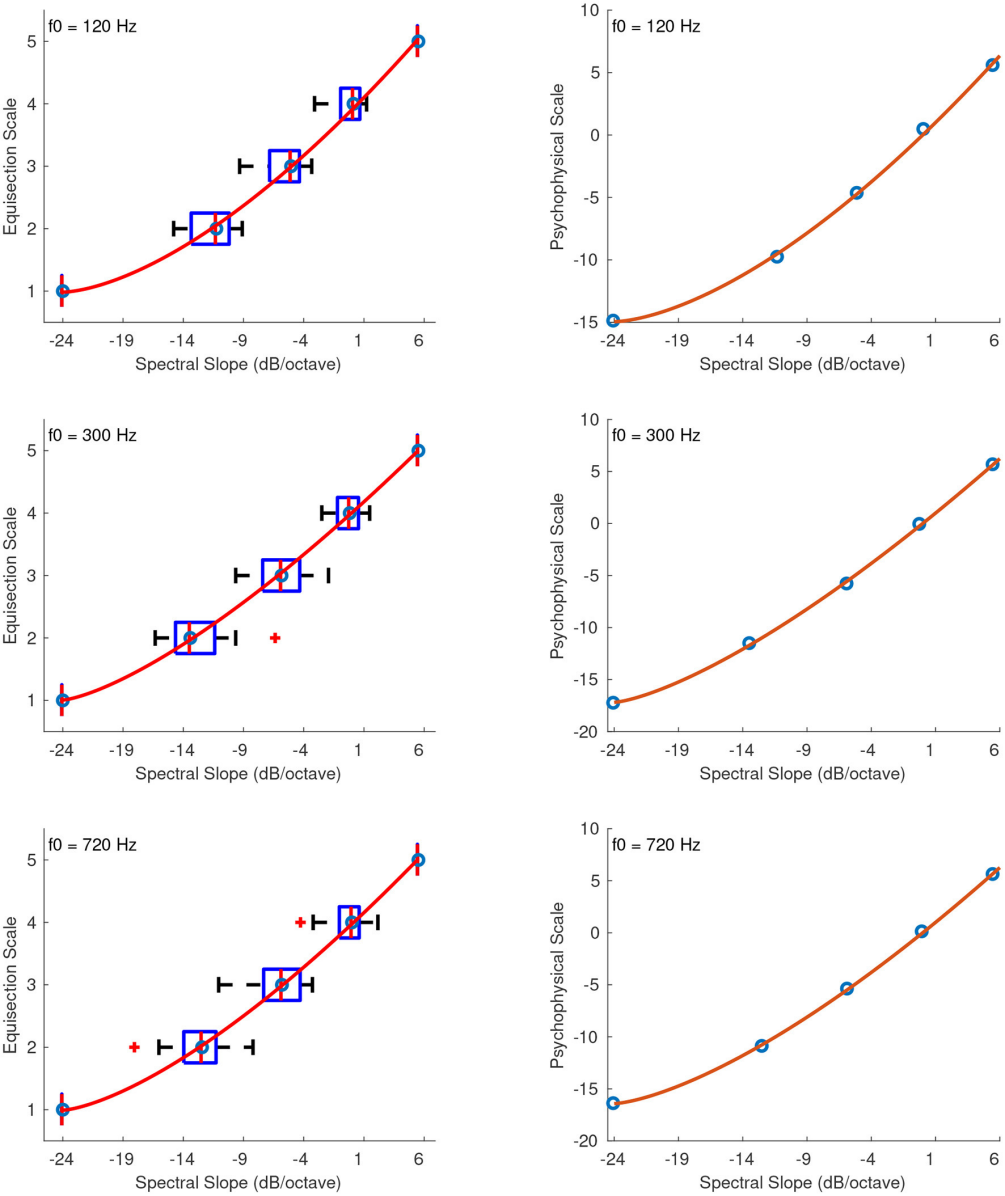
Equisection and psychophysical scales of spectral slope at three f0s. Left panels, boxplots and fitting functions on the median ratings. Whiskers extend to 2.7 SD. Right panels, psychophysical scales and extrapolated fitting functions.

The best-fitting function for the stimuli of spectral slope was again a power function and the stimulus values *x* were rescaled to strictly positive before applying the fitting function according to: *x*′ = *x* + 24, where *x* is the original stimulus value. The rescaling constant of +24 allowed values above –24 dB/octave to be included in the scale. Figure 14 **(left panel)** shows the fitted functions for stimuli at 120, 300, and 720 Hz, on top of the median ratings and interquartile ranges for the actual stimulus values of spectral slope. The right panel of Figure 14 shows the psychophysical scale in the range of –24 dB/octave to +6 dB/octave slope. The zero point of the scale was naturally assigned the value of 0 which corresponds to zero spectral slope, and the units of the scale were derived after assigning a value of 1 to a spectral slope of +1. The final fitting equations for stimuli at 120, 300, and 720 Hz, after the unit assignments are, respectively:


(31) $$\begin{array}{l} f_{120}\left(x^{\prime }\right)=9.673\cdot 10^{-2}{x\prime }^{1.586}-14.95,\;R^{2}=0.99 \end{array}$$

(32) $$\begin{array}{l} f_{300}\left(x^{\prime }\right)=21.06\cdot 10^{-2}{x\prime }^{1.385}-17.19,\;R^{2}=1 \end{array}$$

(33) $$\begin{array}{l} f_{720}\left(x^{\prime }\right)=16.46\cdot 10^{-2}{x\prime }^{1.448}-16.42,\;R^{2}=1 \end{array}$$

### 4.3. Discussion

For almost all features, the reliability of the derived scales within the tested range was excellent as indicated by Cronbach's *alpha*. The equisection of the physical continuum for each feature was performed on stimuli presented in both ascending and descending directions, which controlled for any hysteresis effects on the derived scales. With the exception of spectral skewness, for which the best fitting function on the median (averaged) ratings was a third-order polynomial, the best fitting functions of the rest of the descriptors were all power functions, albeit exhibiting significantly different shapes, indicating that each descriptor is perceived on a different psychophysical scale. At this point, it should also be pointed out that Torgerson's (1958) method, which was used for deriving a single psychophysical scale of spectral spread, would not have been applicable if listeners were not internally consistent. The linearity of the functions used to convert the overlapping scale values of the upper and lower ranges into the values of the middle range indicates that the estimated equisection points were in fact a function of spectral spread and not of the presented range.

With the exception of spectral centroid, for which the zero point of the scale was derived by extrapolating the fitting function, the rest of the scales were assigned a zero point that has a physical meaning and maps naturally to the physical value of the stimulus. For spectral centroid, which is related to the perception of auditory brightness (see, for instance, Schubert and Wolfe, 2006), the zero point was assigned empirically at 20 Hz, which marks the lower limit of pitch perception. Although it can be argued that the centroid will in general have values above 20 Hz, there can still be cases in which the centroid is evaluated on spectra with minimal or zero spectral spread. With respect to such cases, in which the spectral centroid would match the stimulus fundamental frequency, and after taking into account Schubert and Wolfe's (2006) conclusion that brightness is dependent upon f0 to the extent that increasing f0 also increases spectral centroid, it was concluded that frequencies as low as 20 Hz should not be excluded from the psychophysical ratio scale of spectral centroid. The numerical ranges of the scales corresponding to the minimum and maximum physical values of the audio features are all comparable in terms of magnitude, because the unit assignment, albeit arbitrary, was performed in such a way as to facilitate comparisons between different audio features when these are extracted from a given stimulus.

In previous experiments on ordinal scaling (Kazazis et al., 2021), we have shown that the perception of some audio features depends on the fundamental frequency or its spectral centroid, and therefore, with the exception of the scales of spectral centroid and spectral spread, the psychophysical scales for the rest of the features were derived separately for each fundamental frequency or centroid tested. A scale for a particular f0 or spectral centroid that falls in between the tested range of this study, can be derived by using a weighted interpolation scheme between the coefficients of the fitting functions in log-frequency. Of course, that would not have been possible if the fitting functions that were used to derive the ratio scales of a particular feature and at each f0 or centroid were not of the same form.

## 5. Conclusions

The aim of the present study was to test listeners' abilities to estimate intervals of audio features, and the construction of perceptual ratio scales, when each of the presented features was independently controlled through specifically designed synthesis algorithms. The experimental design used in both experiments controlled for the biases of order effects, in which listeners' judgments of a particular stimulus often depend on (1) the preceding stimuli, (2) hysteresis effects in which judgments are biased from the ascending or descending presentations of stimulus values, and wherever possible, (3) for range effects, which occur when listeners' judgments are performed on a limited range of stimulus values. However, for the stimulus sets of spectral centroid, spread, and especially skewness, the range effects were the hardest to control for due to the constraints imposed by the synthesis algorithms. These constraints include: (1) the choice of using nine harmonics at a fixed f0 for keeping the spectral spread and skewness fixed in the centroid stimulus sets, (2) bandwidth restrictions due to fixed f0, centroid, and skewness for the spectral spread stimulus sets, and (3) the narrow permissible range of skewness in the Skew-normal distribution, which was employed to keep the centroid and spread fixed in the skewness stimulus sets.

In the first experiment, listeners made estimations based on successive differences between stimuli of a given audio feature, and thus this experiment provided interval scale measurements. There was a large variability in the reliability of the ratings measured according to Cronbach's *alpha*, which was dependent on the presented spacing of stimuli. The largest biases of the derived interval scales resulted from the centering tendency of the listeners, and for some features, from the marginally supraliminal stimuli used in the combined stimulus set. Despite these biases, the experiment is to be considered successful because in general, the median values of the interval estimations increased monotonically with increasing stimulus value, and thus confirmed the ability of listeners to estimate intervals between stimuli of a given audio feature.

The method of equisectional scaling, which was employed in Experiment 2, leads to equal sensory intervals that have built-in ratio properties and thus, the results of that experiment provided ratio scale measurements. The interval scaling in Experiment 1 was a prerequisite for proceeding to the construction of ratio scales, which was the ultimate goal of this study, because without any prior evidence that listeners were actually capable of estimating intervals of audio features, the results of the second experiment would have been subject to the uncertainty of whether they were visually bisecting or quartering the displayed range of stimulus values, instead of performing estimations according to prescribed psychophysical ratios (i.e., halving and quartering) of auditory stimuli. As evidenced by Cronbach's *alpha*, the reliabilities of the derived psychophysical scales were overall excellent. With the exception of spectral centroid, where the zero point was derived by extrapolating the fitting function, the rest of the zero points of the derived psychophysical scales were mapped naturally to the stimulus physical values, and the units, albeit arbitrary, were assigned to facilitate in a listener's mind any comparisons across the values of different features when these are extracted from a single stimulus. Due to constraints imposed in the synthesis process for independently controlling each tested feature and constructing perceptually uncorrelated stimuli, the extreme values of the psychophysical scales were derived by extrapolating the fitting functions. Nevertheless, although the extrapolation was well behaved (in terms of monotonicity) further experiments are needed to verify the presented scales in the extrapolated regions.

The results of the two experiments are not directly comparable because in the first experiment, the listeners' task was to estimate intervals between successive stimuli, whereas in the second experiment the task was to equisect a given range of each features' continuum, which after the zero point assignment on the fitting function led to ratio scales with internally consistent judgments. In addition, in the first experiment, the reliability scores for some stimulus sequences of a particular feature were considerably lower than the overall excellent reliability observed in the ratio scaling experiment. However, in both experiments, the form of the fitting functions on the interval and ratio estimations of each feature were of the same kind (i.e., power functions), with the exception of spectral skewness. For spectral skewness, although in the first experiment the best fitting function was a fifth-order polynomial, whereas in the second experiment, it was a third-order polynomial, the resultant shapes of both functions highlight the asymmetrical judgments between negative and positive skewness, which were also pointed out in Kazazis et al. (2021).

In most cases, in order to test a wide range of values for each feature, separate scales were derived for each of the fundamental frequencies or spectral centroids used within each stimulus set. If a psychophysical scale for a feature at an intermediate fundamental frequency or centroid is needed, it can be derived by interpolating the coefficients of the fitting functions derived from the present study. However, for sounds having fundamental frequencies or centroids that are located below or above the tested range of this study, the derived scales should be used with caution, as their validity remains speculative before conducting any further experiments.

The construction of psychophysical scales based on univariate stimuli, allowed for the establishment of cause-and-effect relations between audio features and perceptual dimensions, contrary to past research that has relied on multivariate stimuli and has only examined the correlations between the two. Finally, the psychophysical scaling of audio features presented in this study is a prerequisite and essential step before one starts to study timbre as a phenomenon that emerges from a combination of audio features and explore its attributes through perceptual dominance hierarchies of those features.

## Data Availability Statement

The raw data supporting the conclusions of this article will be made available by the authors, without undue reservation.

## Ethics Statement

The studies involving human participants were reviewed and approved by McGill University Research Ethics Board II. The patients/participants provided their written informed consent to participate in this study.

## Author Contributions

SK, PD, and SM designed the study. SK and PD designed the stimulus algorithms. SK collected the data and drafted the manuscript. All authors conducted data analyses, edited, and approved the manuscript.

## Funding

This work was supported by grants from the Canadian Natural Sciences and Engineering Research Council awarded to SM (RGPIN-2020-04022) and to PD (RGPIN- 2018-05662), and as well as a Canada Research Chair (950-231872) awarded to SM.

## Conflict of Interest

The authors declare that the research was conducted in the absence of any commercial or financial relationships that could be construed as a potential conflict of interest.

## Publisher's Note

All claims expressed in this article are solely those of the authors and do not necessarily represent those of their affiliated organizations, or those of the publisher, the editors and the reviewers. Any product that may be evaluated in this article, or claim that may be made by its manufacturer, is not guaranteed or endorsed by the publisher.

## Footnotes

^1^The power function requires that all data points be strictly positive, and therefore the actual value used was 2^−52^ Hz instead of 0 Hz.
